# Advances in Superlattice Hydrogen Storage Alloys: Preparation Method, Phase Structure Modulation, and Hydrogen Storage Performance

**DOI:** 10.3390/molecules30102161

**Published:** 2025-05-14

**Authors:** Yuqing Zhang, Yajie Zhang, Tianmeng He, Hao Wang, Shubin Zhang, Jinpeng Wang, Xiaoyi Xue, Yanrong Liu, Biaohua Chen

**Affiliations:** 1 College of Environmental Science and Engineering, Beijing University of Technology, Beijing 100124, China; zhangyuqing@ipe.ac.cn (Y.Z.); chenbh@bjut.edu.cn (B.C.); 2 CAS Key Laboratory of Green Process and Engineering, State Key Laboratory of Mesoscience and Engineering, Beijing Key Laboratory of Solid State Battery and Energy Storage Process, Institute of Process Engineering, Chinese Academy of Sciences, Beijing 100190, China; haowang@ipe.ac.cn; 3 Longzihu New Energy Laboratory, Zhengzhou Institute of Emerging Industrial Technology, Henan University, Zhengzhou 450000, China; zyj10152022@163.com (Y.Z.); 13526070315@163.com (T.H.); zhangshubin0809@163.com (S.Z.); jinpengwang@henu.edu.cn (J.W.); 4 Energy Engineering, Division of Energy Science, Luleå University of Technology, 97187 Luleå, Sweden; 5 School of Chemical Engineering, University of Chinese Academy of Sciences, Beijing 100049, China

**Keywords:** superlattice hydrogen storage alloys, rare-earth element doping, element substitution, phase structure, hydrogen storage capacity

## Abstract

Superlattice hydrogen storage alloys have attracted much attention due to their high capacity, excellent cyclic stability, and moderate operating conditions. This review, focusing on journal articles published between 2020 and 2025, comprehensively covers the impacts of doping with different rare-earth elements and the substitution of different elements on superlattice hydrogen storage alloys and details the influence mechanisms of different preparation methods, such as arc melting and powder metallurgy, on the phase structure of alloys. A thorough analysis is conducted on how rare-earth element doping alters the crystal structure, lattice parameters, and phase stability of alloys, thereby affecting their hydrogen storage performance. Meanwhile, the differences in the effects of different substituting elements at various substitution sites on the phase structure and hydrogen storage performance of alloys are explored, and the regular patterns and influencing factors are summarized. This review provides a new perspective for the design and development of high-performance superlattice hydrogen storage alloys and is expected to contribute to the long-term and sustainable development of clean hydrogen energy.

## 1. Introduction

The rapid growth of the population and economic development have accelerated the use of fossil fuels and the worsening of environmental pollution. Hydrogen, as a green, clean, efficient, and non-polluting secondary energy source, can become an effective alternative to traditional fossil fuels [1,2,3,4]. Moreover, the current global push for decarbonization and the demand for clean energy make green hydrogen the fuel of the future. In the development of hydrogen energy technology, efficient hydrogen storage represents a crucial stage in the utilization of hydrogen energy. Common techniques for hydrogen storage encompass high-pressure compression, liquefaction at low temperatures, and solid-state adsorption [5,6,7,8,9,10,11,12,13,14]. Solid-state hydrogen storage stands out as an optimal method compared to others, owing to its benefits, such as reduced storage pressure, high volumetric density, and enhanced safety [15,16,17].

The capacity for hydrogen storage and release in materials plays a pivotal role in the advancement of solid-state hydrogen storage technology. Consequently, the research and development of high-performance hydrogen storage materials have garnered significant attention. Over the past decades, scientists have invented numerous hydrogen storage materials, including hydrogen storage alloys such as those based on rare-earths [18], titanium [19], and vanadium [20], as well as complex metal hydrides of metal borohydrides and nitrides [11,21,22,23]. Additionally, adsorption materials, including carbon-based materials and metal-organic frameworks (MOFs) [24,25,26], have also been extensively explored. Currently, only hydrogen storage alloy materials have been widely applied in practical use. According to the different elemental compositions and structures, hydrogen storage alloys mainly contain AB_5_ [27,28,29,30,31], AB_2_ [18,32,33,34,35], AB [36,37,38], A_2_B [39,40], vanadium-based BCC [19,20,41,42], AB_3-4_ [43,44,45,46,47], etc., where A refers to the element that contributes to the hydrogen storage capacity, and B refers to the element that plays a catalytic role in regulating the adsorption and desorption performance of hydrogen storage. AB_5_-type hydrogen storage alloy is currently a commercially available hydrogen storage material, but its low hydrogen storage capacity limits its development. While the AB_2_-type hydrogen storage alloy boasts a higher capacity than the AB_5_-type, it necessitates a prolonged activation period [48].

In recent years, superlattice hydrogen storage alloys, such as AB_3-4_ (including AB_3_, A_2_B_7_, A_5_B_19_, and AB_4_) [49,50,51,52,53,54], have garnered considerable attention because of their combination of favorable activation performance akin to AB_5_-type structures and high hydrogen storage capacity, which is similar to AB_2_-type structures. These alloys are usually formed by stacking two substructure units, [AB_5_] and [A_2_B_4_], along the C-axis. Numerous studies have indicated that the phase structure and composition significantly influence the performance characteristics of superlattice hydrogen storage alloys. For example, Wang et al. [55] prepared hydrogen storage alloys with multiphase and 2H-type A_2_B_7_ single-phase structures, tested their performance, and found that single-phase alloys have better hydrogen storage and release capacity. Therefore, understanding the correlation between the phase transition process and the performance of superlattice hydrogen storage alloys is crucial for further improving their hydrogen storage capacity. Moreover, the complex stacking method of superlattice hydrogen storage alloys has a negative impact on their cyclic stability. During the process of hydrogen absorption and desorption, the [A_2_B_4_] and [AB_5_] sublattices undergo asynchronous expansion and contraction, leading to significant microstrain at their interfaces and subsequent lattice structure damage. This results in a diminished hydrogen storage capacity and poor cycling stability [56]. Hence, achieving a balance between the volume dimensions of these two sublattices is crucial for enhancing the cycling stability of hydrogen storage alloys.

In the pursuit of efficient hydrogen storage materials for advancing hydrogen-based energy technologies, superlattice alloys stand out compared to conventional metal hydrides, particularly in terms of their reversible capacity and kinetic performance. Conventional metal hydrides face notable limitations. Mg-based hydrides like MgH_2_ and Mg_2_NiH_4_ are hindered by strong Mg-H bonds [57]. Breaking these bonds during dehydrogenation is arduous, necessitating extremely high temperatures of 250–400 °C. This not only consumes substantial energy but also raises concerns about the system’s long-term stability and safety. Meanwhile, complex hydrides, such as borohydrides [58], despite offering high hydrogen storage potential, suffer from irreversible phase segregation. During hydrogen absorption–desorption cycles, their complex crystal structures break into distinct phases that fail to recombine upon re-hydrogenation, leading to a gradual decline in storage capacity over multiple cycles and rendering them impractical. The confined lattice transitions in superlattice alloys, where the alternating layers restrict atomic movement and rearrangement during hydrogenation and dehydrogenation, enable more efficient and reversible hydrogen release at moderate temperatures. This allows them to operate within a practical temperature range, avoiding the extreme conditions required by conventional metal hydrides. Consequently, superlattice alloys significantly reduce energy consumption and enhance the feasibility of hydrogen storage systems, as clearly demonstrated by the detailed performance data in Table 1. Moreover, the high cost associated with these alloys is a major hurdle, largely because they often contain scarce and expensive rare-earth elements such as lanthanum and cerium. Their limited global reserves and volatile prices drive up production expenses. In terms of performance, they exhibit poor high-temperature stability, with their hydrogen storage capabilities deteriorating at elevated temperatures. Additionally, these alloys are prone to powdering as a result of substantial volume expansion during hydrogen uptake, which further reduces their cycle durability.

**Scheme 1:**
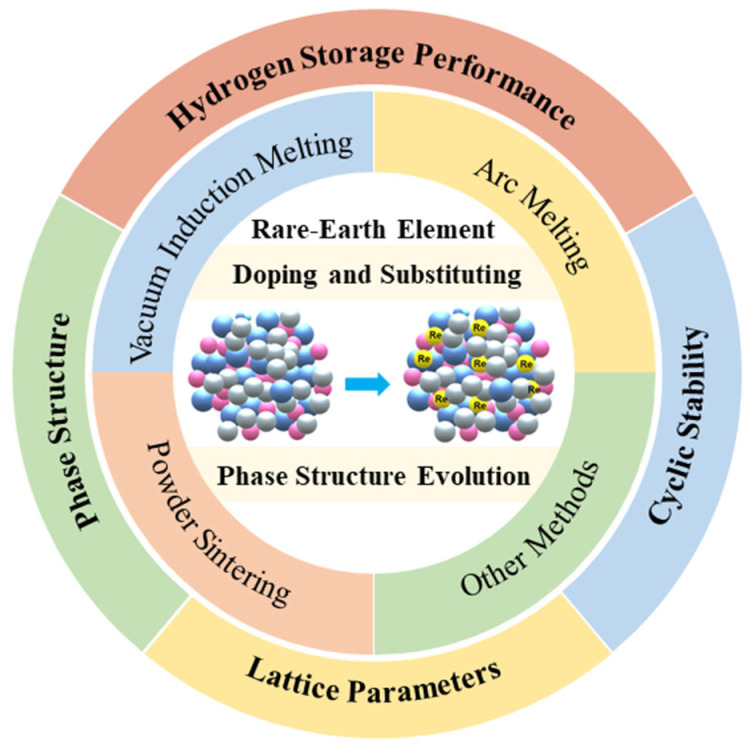
The framework of this review.

Investigating how preparation methods and doping/substitution elements affect cell volume and phase structure, and subsequently understanding their influence on alloy hydrogen storage performance, is advantageous for designing and developing advanced hydrogen storage materials. However, there are currently few systematic summaries of hydrogen storage in superlattices. This review concentrates on journal publications from the last five years and summarizes the relationship between preparation techniques and the phase structure of superlattice hydrogen storage alloys, along with the effects of elemental doping/substitution on their phase structure and properties. Scheme 1 shows the overall framework of this review. Such insights offer valuable direction for the purposeful design of optimal hydrogen storage materials.

## 2. Preparation Methods of Superlattice Hydrogen Storage Alloys

Various experimental methods have been developed to study superlattice hydrogen storage alloys, including vacuum induction melting, arc melting, powder metallurgy, etc. [59,60,61,62,63]. Among them, vacuum induction melting is the most commonly used preparation method, and powder metallurgy is the most effective method for preparing single-phase alloys. In this section, we will focus on the alloy phase transformation and composition of alloys obtained by different preparation methods.

### 2.1. Vacuum Induction Melting Method

**Figure 1:**
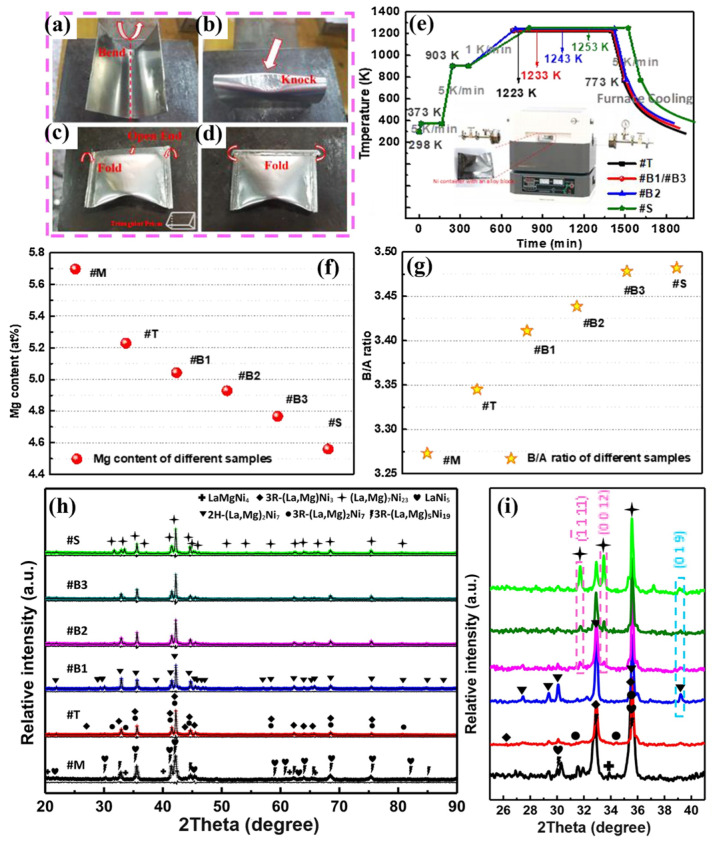
(**a**–**d**) Preparation stages of the nickel container; (**e**) heat treatment equipment and its procedures; (**f**) Mg contents and (**g**) B/A ratios of the alloy samples. XRD patterns in the 2*θ* range of (**h**) 20–90 degrees and (**i**) 25–41 degrees of the #M, #T, #B1–#B3, and #S alloys. Adapted with permission from Ref. [63]. Copyright 2021, copyright Liu, J et al.

Induction melting stands out as a prevalent method for the preparation of superlattice hydrogen storage alloys (Table 2). The superlattice alloys prepared by induction melting are usually multiphase. For example, Du et al. [64] prepared La_1−x_Y_x_Mg_0.3_Ni_3.5_ (x = 0.00, 0.14, 0.28, 0.35)-series alloys, all of which have a three-phase structure. Lim et al. [65] prepared La_(0.65−x)_Ce_x_Ca_1.03_Mg_1.32_Ni_(9−y)_Al_y_ (x = 0, 0.25, 0.5; y = 0, 0.25) alloys by a combination of arc and induction melting. The obtained alloys are all multiphase alloys, among which the alloy without Ce and Al substitution contains only AB_3_ and AB_5_ phases. However, annealing of the resulting alloy can significantly reduce its phase composition and even result in a single-phase structure. Through precisely controlling the initial alloy composition, heat treatment procedure, and magnesium volatilization, Liu et al. [63] successfully prepared the La_0.60_Pr_0.15_Mg_0.25_Ni_3.25_ alloy and investigated the influence of the annealing process on its crystalline phase (Figure 1). The results indicated that the melted sample comprised six distinct crystal phases, and annealing significantly enhanced the composition and content of these crystal phases within the alloy. As the annealing temperature was increased to 1253 K, the alloy transformed into an A_2_B_7_ single-phase structure (Figure 1h,i). The specific phase transition process is as follows: at 1223 K, LaMgNi_4_ decomposes and reacts with LaNi_5_ and 3R- (La, Mg)_5_Ni_19_ to form 2H- (La, Mg)_2_Ni_7_, and then 3R- (La, Mg) Ni_3_ further reacts with LaNi_5_ and 3R- (La, Mg)_5_Ni_19_ to form 2H- (La, Mg)_2_Ni_7_; at 1233 K, 3R- (La, Mg)Ni_3_ decomposes and reacts with 3R- (La, Mg)_2_Ni_7_ to form (La, Mg)_7_Ni_23_; at 1243 K, some (La, Mg)_7_Ni_23_ phases transform into 2H- (La, Mg)_2_Ni_7_ phases; the A_2_B_7_ single-phase structure is obtained when the temperature rises to 1253 K. Liu et al. [48] prepared La_0.75−x_Gd_x_Mg_0.25_Ni_3.5_ (x = 0, 0.05, 0.1, 0.15) alloys using the induction melting method and obtained a single-phase 2H-type A_2_B_7_-phase structure by adjusting the annealing temperature of different Gd substitute alloys. Lu et al. [66] prepared a La_0.60_Sm_0.20_Mg_0.20_Ni_3.50_Al_0.20_ hydrogen storage alloy with the AB_4_ phase as the main phase using La, Sm, Mg, Ni, and Al metals as raw materials through induction melting and annealing processes. It was found that the AB_4_ phase was formed between 970 °C and 1000 °C through the transgranular reaction of the A_5_B_19_ phase and CaCu_5_ phase. The prepared alloy also exhibited good resistance at low temperatures (−40 °C). Furthermore, the phase composition of the alloy could be modulated through elemental doping or substitution. As demonstrated in the research of Zhou et al. [67], an increase in the Y doping content leads to a gradual decrease in the abundance of the AB_3_ phase.

### 2.2. Arc Melting

**Figure 2:**
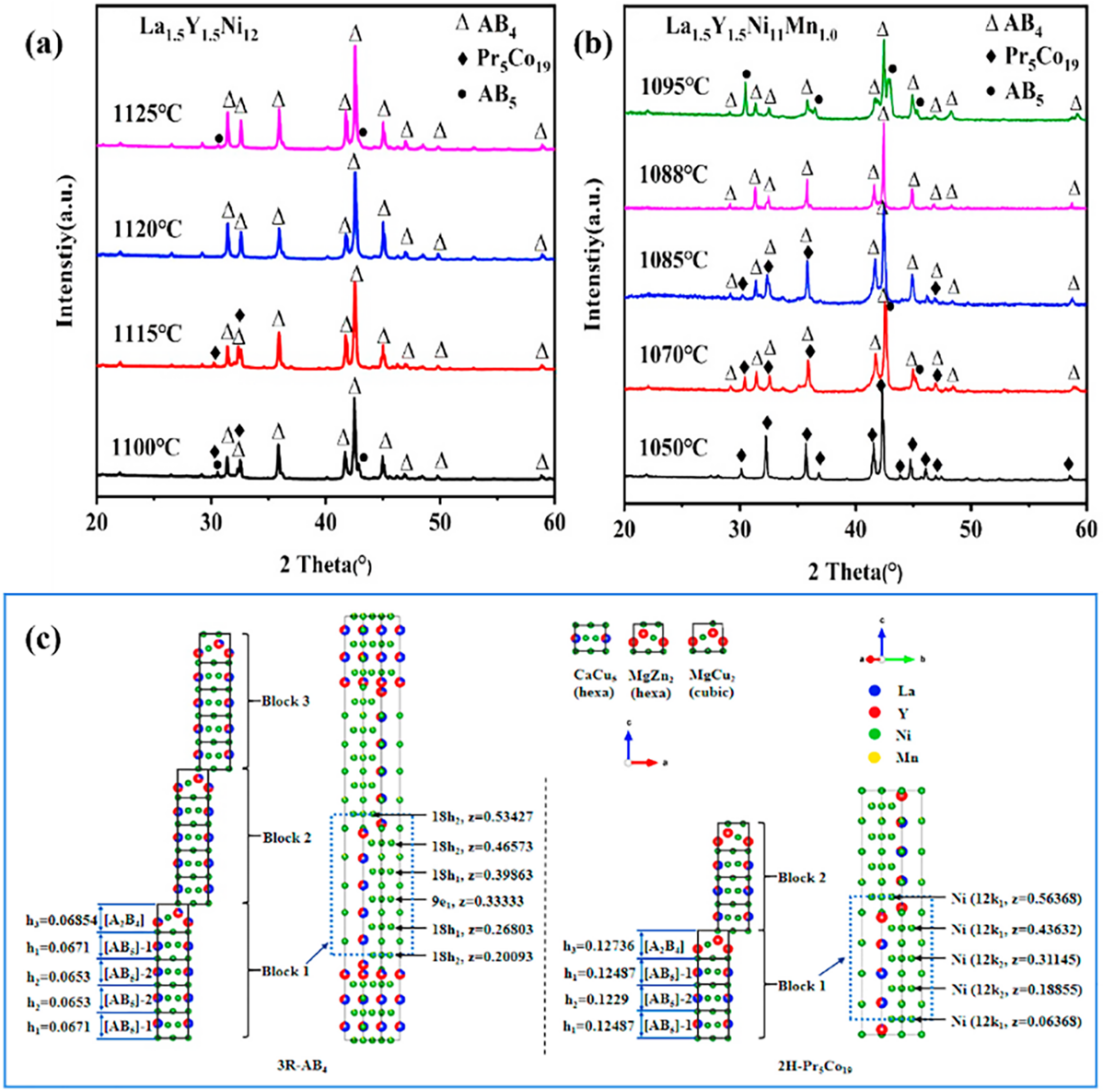
XRD patterns of (**a**) La_1.5_Y_1.5_Ni_12_ and (**b**) La_1.5_Y_1.5_Ni_11_Mn_1.0_ alloys annealed at different temperatures; (**c**) illustrative diagrams depicting the 2H-Pr_5_Co_19_-type and 3R-AB_4_-type structural configurations within the La_1.5_Y_1.5_Ni_11_Mn_1.0_ alloy. Adapted with permission from Ref. [73]. Copyright 2023, copyright Wan, C et al.

In addition to induction melting, the combination of arc melting and annealing processes can be used for the controllable preparation of hydrogen storage alloys (Table 3). Iwase et al. [69] prepared Pr_5_Ni_19_ alloy using the arc melting method with Pr and Ni as raw materials and obtained 2H-type A_5_B_19_ single-phase alloy at an annealing temperature of 1403 K. Afterwards, they prepared GdNi_3_ alloy and Nd_2_Ni_7_ alloy using the same method, and obtained GdNi_3_ alloy with the PuNi_3_ phase as the main phase at a temperature of 1223 K [70] and Nd_2_Ni_7_ alloy with the Ce_2_Ni_7_ phase as the main phase at 1448 K [71]. Neither of these alloys achieved a single-phase structure within the controlled temperature range. Zhao et al. [72] obtained Gd_2_Co_7_ single-phase LaY_2_Ni_10.5_ alloy and Ce_2_Ni_7_ single-phase LaY_2_Ni_10.5_ alloy by annealing at 1000 °C and 1100 °C, respectively. Wan et al. [73] obtained the 3R-AB_4_ single-phase structure for the first time in a La-Y-Ni-based system through annealing temperature control. As shown in Figure 2, for La_1.5_Y_1.5_Ni_12_ alloy, the temperature at which a single-phase structure is obtained is 1120 °C. When the temperature is slightly increased or decreased, the obtained alloy contains secondary phases; The La_1.5_Y_1.5_Ni_11_Mn_1.0_ alloy also showed similar results, with a single-phase structure obtained at a temperature of 1088 °C. Therefore, the 3R-AB_4_ phase is characterized as a high-temperature phase that exhibits a narrow annealing temperature window. In this preparation method, element substitution also affects the alloy phase content and structure. By modifying the Al content, Wan et al. [74] synthesized (La_0.33_Y_0.67_)_5_Ni_18.1−x_Mn_0.9_Al_x_ (x = 0, 0.3, 0.6, 0.9) alloys. The primary phase in all these alloys is the 2H-Pr_5_Co_19_-type phase. As the Al content increases, the abundance of this phase initially decreases but then rises again.

### 2.3. Powder Sintering Method

**Figure 3:**
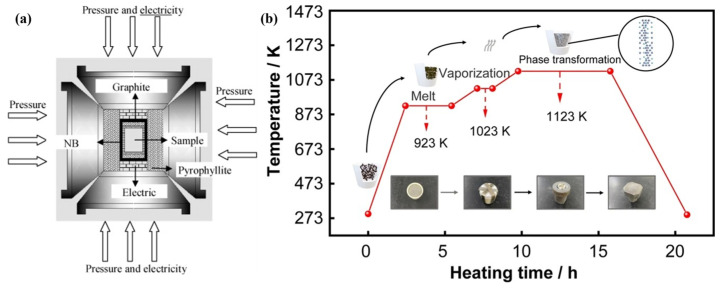
(**a**) Diagrammatic representation of the high-pressure sintering equipment; Reprinted with permission from Ref. [76]. Copyright 2013, copyright Liu, J et al. (**b**) heating procedures of Mg thermal diffusion treatment. Reprinted with permission from Ref. [77]. Copyright 2024, copyright Zhang, Y.-X et al.

The powder sintering method is the most effective way of producing single-phase alloys, and the preparation process usually includes several steps, such as precursor crushing, mixing, pressing, and sintering. In powder sintering, researchers usually regulate the types and ratios of precursors to obtain alloys with different phase structures (Table 4). For example, Liu et al. [56] obtained La_2_MgNi_9_ alloy with the 3R-type AB_3_ single phase, La_3_MgNi_14_ alloy with the 2H-type A_2_B_7_ single phase, and A_5_B_19_-phase La_4_MgNi_19_ alloy with the coexistence of 2H and 3R types by regulating the ratio of LaMgNi_4_ to LaNi_5_. Zhao et al. [75] obtained alloys with single-phase CaCu_5_, PuNi_3_, Ce_2_Ni_7_, and Pr_5_Co_19_ structures by adjusting the ratio of LaMgNi_4_ to La_0.60_Gd_0.15_Mg_0.25_Ni_3.60_. In addition to the conventional powder sintering method, researchers have conducted research on other sintering processes. Liu et al. [76] used high-pressure sintering to obtain La_0.75_Mg_0.25_Ni_3.50_ alloy by adjusting the pressure during the sintering process (1.5–4.0 GPa) (Figure 3a). During sintering at pressures ranging from 1.5 to 2.5 GPa, the alloy primarily consists of Ce_2_Ni_7_ and Pr_5_Co_19_ phases, with LaNi_5_ as a secondary phase. However, at 4.0 GPa, the MgNi_2_ phase emerges, accompanied by an increase in the abundance of the LaNi_5_ phase and a decrease in the Ce_2_Ni_7_ and Pr_5_Co_19_ phases. Utilizing the thermal diffusion sintering technique, Zhang et al. [77] synthesized Mg (x)/La_0.74_Sm_0.03_Y_0.23_Ni_4.32_Al_0.04_ (1 − x) (x = 0, 0.17, 0.21, 0.28, 0.33, 0.38) alloys (Figure 3b). Raw materials with different Mg contents were mixed with a La_0.74_Sm_0.03_Y_0.23_Ni_4.32_Al_0.04_ precursor, sealed in a small crucible, and sintered at 1123 K for 6 h. The researchers discovered that as the Mg content rises, the LaNi_5_ phase present in the initial alloy diminishes. Specifically, at x = 0.28, the crystalline structure undergoes a transformation, resulting in the formation of Gd_2_Co_7_ and Ce_2_Ni_7_ phases. With a further increase in Mg content (x = 0.38), a MgCu_4_Sn phase is formed. At x = 0.28, the alloy exhibits optimal hydrogen storage performance.

### 2.4. Spark Plasma Sintering

Spark plasma sintering (SPS) is a new sintering technology featuring rapid sintering, low-temperature densification, high-efficiency particle surface activation, precise control of process parameters, and broad material applicability. For instance, Zhang et al. [78] prepared a new ternary La_0.85_Mg_0.15_Ni_3.8_ via SPS under 50 MPa and at temperatures from 810 °C to 900 °C, using Mg_2_Ni and LaNi_y_ (y = 4.33) as precursors during the preparation process. The Mg_2_Ni phase is liquid in this temperature range, which causes partial magnesium loss due to sublimation. To ensure the final sample met the target composition (La_0.85_Mg_0.15_Ni_3.8_), the initial material composition was optimized through a twofold adjustment. Excess Mg_2_Ni was added to compensate for magnesium loss at high temperatures, while the nickel content in the LaNi_y_ system was proportionally reduced (with the composition of LaNi_y_ maintained at y = 4.33). This synergistic adjustment strategy effectively preserved the stoichiometric ratio of the target composition. The SPS technique, by precisely controlling the phase composition (such as stabilizing the 1: 4 phase at high temperatures), inhibiting magnesium volatilization, and rapidly forming a homogeneous structure, enables the La_0.85_Mg_0.15_Ni_3.8_ alloy to exhibit a stable, moderate hydrogen storage capacity (1.3 wt%) and a platform pressure suitable for battery applications (0.08 MPa).

### 2.5. Mechanical Alloying

Mechanical alloying (MA) is a technology that achieves the solid-state alloying of powders through high-energy ball milling. Its main advantages include breaking through the limitations of traditional melting to enable the preparation of unconventional alloys, refining grain size, and regulating microstructure; its wide applicability and flexible processing; and its simple process and controllable cost. Nowak et al. [52] carried out mechanical alloying for 48 h on accurately weighed and thoroughly mixed elemental powders in an argon atmosphere using 12 mm diameter hard-steel balls. During the mechanical alloying process, the impact of the steel balls caused the powders to continuously fracture, mix, and cold-weld. This not only reduced the powder particle size but also triggered chemical reactions between the elements, thus forming an alloy precursor with specific structures and properties. The materials obtained after mechanical alloying were annealed at 1123 K for 0.5 h in a high-purity argon environment to relieve the internal stress of the alloy, promote the uniform diffusion of elements, contribute to the formation of a more stable crystal structure, and further optimize the performance of the alloy. The as-prepared alloys have a multiphase structure, including phases such as the La_2_Ni_7_, CaCu_5_, and La_2_O_3_ types. The mass fraction of the A_2_B_5_-type phase decreases from 92.3% to 60.0% with the increase in Gd content. The La_1.25_Gd_0.25_Mg_0.5_Ni_7_ alloy has the highest content of the La_2_Ni_7_ phase, and the elements are uniformly distributed. All the alloys can absorb hydrogen at 303 K, and the pressure of the absorption and desorption plateau is related to the Gd content. An increase in the Gd content leads to an increase in the hydrogen absorption pressure. Except for the La_1.25_Gd_0.25_Mg_0.5_Ni_7_ alloy, the hydrogen absorption kinetics of most alloys improve after two cycles. The La_1.25_Gd_0.25_Mg_0.5_Ni_7_ alloy takes the shortest time (4 min) to reach 95% of its maximum hydrogen absorption capacity. The substitution of Gd reduces the maximum hydrogen storage capacity.

### 2.6. Additive Manufacturing

Additive manufacturing technologies, especially laser powder bed fusion (L-PBF) technology, exhibit numerous advantages in alloy synthesis, such as achieving microstructure refinement, obtaining unique microstructures, and regulating microstructure and properties. Taking the fabrication of AlCoCrFeNi_2.1_ EHEA samples layer by layer using a commercial M290 (EOS) L-PBF machine as an example [79], during the printing process, the laser scans rapidly, causing the powder to melt and solidify layer by layer, forming an alloy with a specific microstructure. This method generates a large temperature gradient and rapid cooling (cooling rate: 10^5^–10^7^ K s^−1^), which promotes the formation of a dual-phase nanolamellar structure in the alloy. Traditional methods for preparing AlCoCrFeNi_2.1_ EHEA, such as casting, directional solidification, and thermomechanical treatment, result in alloys having a lamellar thickness in the micrometer or submicrometer range (λ ≈ 0.77–5 μm). In contrast, the lamellar thickness of alloys prepared using L-PBF technology is significantly thinner: the thickness of bcc nanolamellae is 64 ± 24 nm, the thickness of fcc nanolamellae is 151 ± 39 nm, and the corresponding interlamellar spacing is 215 nm—approximately half of that in the starting powder feedstock. Meanwhile, traditional alloys are composed of ordered L1_2_ and B2 phases and contain numerous nanoprecipitates. The alloys prepared by the L-PBF method are fcc and bcc solid solutions without precipitates, showing obvious differences in elemental distribution and phase structure.

### 2.7. Other Methods

**Figure 4:**
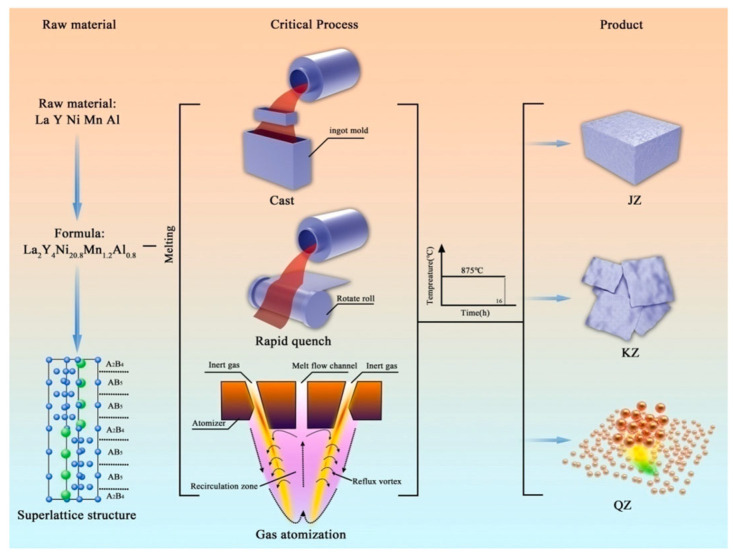
Preparation scheme of the La_2_Y_4_Ni_20.8_Mn_1.2_Al_0.8_ HSAs. Reprinted with permission from Ref. [61]. Copyright 2025, copyright Zhou, S et al.

In addition to the commonly used methods for preparing superlattice alloys mentioned above, researchers have also made other attempts (Table 5). Zhou et al. [61] prepared La_2_Y_4_Ni_20.8_Mn_1.2_Al_0.8_ alloy using three methods—ingot casting, rapid quenching, and gas atomization—and annealed it under the same conditions (Figure 4). The results showed that the alloys prepared by the three methods all had multiphase structures, among which the alloy produced by the gas atomization method had a spherical structure and a long cycle life. Xu et al. [80] fabricated La_0.85_Mg_0.15_Ni_2.65_Co_1.05_M_0.1_ (M = Zr, Cr, Al, Mn, Ni) alloy using the suspension melting technique, followed by annealing. Their findings revealed that alloys with Ni substitution are predominantly composed of the single-phase A_5_B_19_ structure. Furthermore, the inclusion of Mn or Cr, and Al or Zr, was found to enhance the formation of the Pr_5_Co_19_ phase and the (La, Mg)_2_Ni_7_ phase, respectively. Wang et al. [55] prepared a series of LaY_1.9_Ni_10_Mn_0.5_Al_0.2_ alloys using vacuum induction quenching and annealing process and found that they had a 2H-type A_2_B_7_ single-phase structure at an annealing temperature of 1050 °C.

In summary, we found that there are two methods for obtaining single-phase alloys: powder metallurgy and other preparation methods combined with annealing processes. The regulation of annealing temperature is the main way to achieve phase transformation in superlattice hydrogen storage alloys, and element doping or substitution mainly affects phase abundance. The effective combination of the raw material ratio, element doping/substitution, and the annealing process is expected to achieve controllable preparation of superlattice alloys. In the comparison of the preparation processes of superlattice hydrogen storage alloys, vacuum induction melting and powder metallurgy achieve single-phase structures through vacuum environment inhibition of oxidation, electromagnetic stirring, or the coordinated regulation of particle size and sintering parameters. Their annealing processes have a relatively high temperature tolerance (±25–30 °C) that can be completed through single-stage or gradient annealing, and the failure mode is mainly slow abnormal grain growth. Suitable for the industrial preparation of highly stable hydrogen storage materials, arc melting relies on ultra-high cooling rates to freeze metastable single-phase conditions. However, the annealing temperature window is extremely narrow (±5–8 °C), and multi-stage annealing is required to suppress the precipitation of secondary phases; otherwise, it is prone to cause a sharp decline in hydrogen storage performance. It is more suitable for theoretical capacity development but requires in situ monitoring or nanocrystalline technology to break through process bottlenecks. The differences in thermal stability, annealing complexity, and failure mechanism among the three types of methods provide a clear direction for the selection of application scenarios and the process optimization of hydrogen storage alloys.

## 3. Thermodynamics and Kinetics

In practical research, measuring hydrogen absorption isotherms at different temperatures typically involves using a pressure–composition–temperature (PCT) tester. By introducing hydrogen gas into alloy samples at various temperatures and recording the equilibrium pressure and hydrogen absorption capacity, hydrogen absorption isotherms can be obtained. The Van’t Hoff equation and Arrhenius equation provide important theoretical foundations for studying the hydrogen storage properties of alloys. Through experimental measurements and data analysis, the thermodynamic and kinetic characteristics of alloys can be deeply understood, offering guidance for the design and optimization of alloys.

The Van’t Hoff equation describes the relationship between equilibrium pressure and temperature, essentially reflecting the thermodynamic properties of the alloy during the hydrogen absorption process. The heat of hydride formation (ΔH) and entropy change (Δ*S*) of the alloy can be measured using the absorption isotherms at different temperatures according to the Van’t Hoff equation at thermodynamic equilibrium:

*lnP*_eq_ = −ΔH/*RT* + Δ*S*/*R* (1)

where *T* is the absolute temperature, *R* is the gas constant, and *P*_eq_ is the equilibrium pressure.

The activation energy, *E*_a_, is the energy barrier that needs to be overcome for a reaction to proceed. In the hydrogen absorption process of alloys, the activation energy determines the rate of the hydrogen absorption reaction. The higher the activation energy, the more difficult it is for the reaction to proceed, requiring a higher temperature or pressure to achieve the same reaction rate. The associated activation energies were calculated using the Arrhenius equation:

*lnk* = *lnA* − *E*_a_/*RT* (2)

where *k* is the rate constant, *A* is the pre-exponential factor, *E*_a_ is the activation energy, *T* is the temperature, and *R* is the universal gas constant.

## 4. Single-Phase Superlattice Hydrogen Storage Alloys

In general, the main types of superlattice hydrogen storage alloys are AB_3_, A_2_B_7_, A_5_B_19_, and AB_4_ types, whose crystal structures can be viewed as stacked by two subunits, [AB_5_] (CaCu_5_ type) and [A_2_B_4_], in different ratios along the *c*-axis cycle. This is because the [A_2_B_4_] subunit is divided into two different types, the C14 (MgZn_2_) and C15 (MgCu_2_) types. The superlattice structure consisting of CaCu_5_- and C14-type lattices belongs to the P63/mmc space group, called 2H. On the other hand, the superlattice composed of CaCu_5_ and C15-type lattices has the R$\bar{3}$m space group, referred to as 3R. The alloys of type A_5_B_19_ can be classified into Ce_5_Co_19_ (3R) and Pr_5_Co_19_ types (2H); the alloys of the type A_2_B_7_ can be classified into Gd_2_Co_7_ (3R) and Ce_2_Ni_7_ types (2H); AB_3_-type alloys can be divided into PuNi_3_ (3R) and CeNi_3_ types (2H). Single-phase alloys have a single-phase structure, which avoids the complex interactions between different phases in multiphase alloys and reduces the instability of properties caused by the complex phase structure.

**Figure 5:**
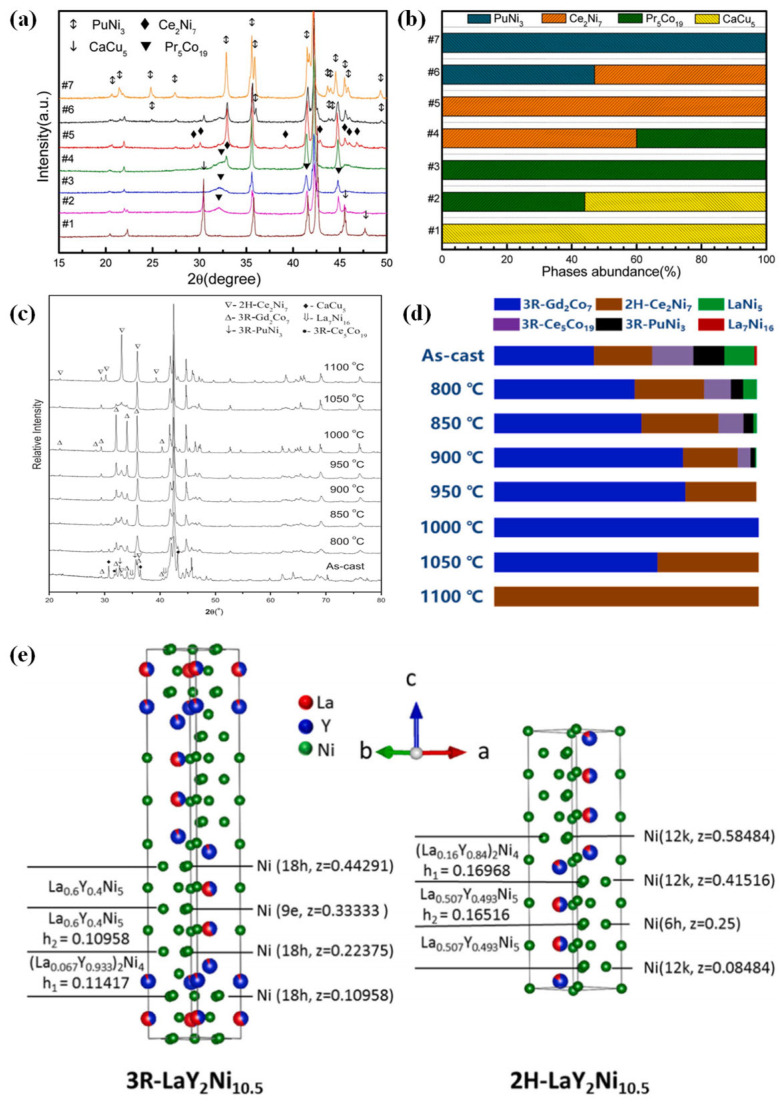
(**a**) The XRD patterns of the La-Gd-Mg-Ni alloy samples; (**b**) the specific phase abundances of the La-Gd-Mg-Ni alloy samples; Adapted with permission from Ref. [75]. Copyright 2020, copyright Zhao, S et al. (**c**) XRD patterns for LaY_2_Ni_10.5_ alloy as-cast and annealed at different temperatures; (**d**) diagram illustrating the variation in phase abundance of LaY_2_Ni_10.5_ alloy with annealing temperature; (**e**) schematic diagram showing the stacking structure of the 3R- and 2H-LaY_2_Ni_10.5_ alloys. Adapted with permission from Ref. [72]. Copyright 2021, copyright Zhao, S et al.

Single-phase alloys serve as excellent models for exploring the relationship between alloy structure and properties, given their straightforward composition. Zhao et al. [75] synthesized La-Gd-Mg-Ni-based hydrogen storage alloys with different superlattice configurations through powder sintering. Their results indicated that a precursor ratio (LaMgNi_4_/La_0.60_Gd_0.15_Mg_0.25_Ni_3.60_) of 1.00 led to the formation of a single-phase structure resembling Pr_5_Co_19_. As the ratio increased to 1.4 and 1.8, the alloys adopted Ce_2_Ni_7_-type and PuNi_3_-type structures, respectively. The Pr_5_Co_19_ type alloys exhibited the highest exchange current density and high multiplicity discharge capability of 769 mAh g^−1^ and 56%, respectively (Figure 5a,b). From the Pr_5_Co_19_ type to PuNi_3_, the increased proportion of [A_2_B_4_] subuniting improves the hydride stability but reduces the charge transfer rate and hydrogen diffusion energy, leading to a decrease in HRD performance. Each element within the alloy contributes uniquely, and an optimal elemental composition can lead to a synergistic effect, bolstering the alloy’s structural stability and enhancing both its charge transfer efficiency and hydrogen storage capacity.

**Figure 6:**
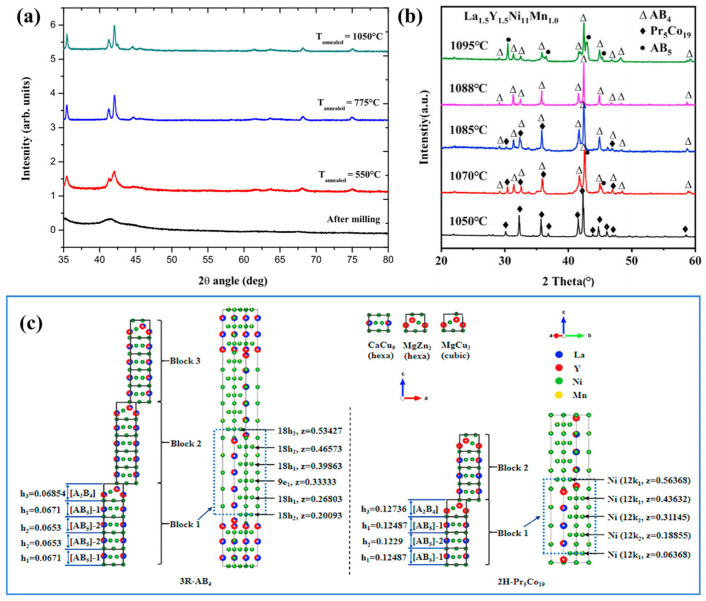
(**a**) XRD patterns of Pr_5_Co_19_ compound as-milled, annealed at 550 °C, 775 °C, and 1050 °C; Reprinted with permission from Ref. [81]. Copyright 2020, copyright Bouzidi, W et al. (**b**) XRD patterns of La_1.5_Y_1.5_ Ni_11_Mn_1.0_ alloy annealed at different temperatures; Reprinted with permission from Ref. [73]. Copyright 2023, copyright Wan, C et al. (**c**) schematic diagrams showing the 2H-Pr_5_Co_19_-type and 3R-AB_4_-type structures in La_1.5_Y_1.5_ Ni_11_Mn_1.0_ alloy. Reprinted with permission from Ref. [73]. Copyright 2021, copyright Wan, C et al.

Precise control of the annealing temperature can be used to adjust the phase structure of the alloy and enhance the alloy properties. Zhao et al. [72] annealed the LaY_2_Ni_10.5_ superlattice alloy at different temperatures. Annealing at 1000 °C resulted in the formation of a pure Gd_2_Co_7_-type (3R) structure, whereas annealing at 1100 °C yielded a Ce_2_Ni_7_-type (2H) structure (Figure 5c–e). The Ce_2_Ni_7_ phase, characterized by a relatively compact [A_2_B_4_] substituent volume of 88.98 Å^3^, demonstrated superior structural stability during charge/discharge cycles. It exhibited a hydrogen discharge capacity of 1.29 wt% and a discharge capacity reaching 219.9 mAh g^−1^. However, the disparity between the [A_2_B_4_] and [AB_5_] substituent volumes caused stress accumulation during cycling, accelerating chalking and corrosion processes. The Gd_2_Co_7_ phase has a more brittle structure but slower capacity decay. Bouzidi et al. [81] found that the nanocrystalline Pr_5_Co_19_ maintains a single 3R structure over a wide range of temperatures (550–1050 °C), which inhibited chalking during hydrogen cycling and prolongs the material life (Figure 6a). Wan et al. [73] used arc melting to prepare La_1.5_Y_1.5_Ni_11_Mn_1.0_ alloy by arc melting. By precisely controlling the annealing temperature (1088 °C) (Figure 6b,c), a pure 3R-AB_4_ phase can be obtained, avoiding the fluctuation in properties caused by the coexistence of multiple phases. The single-phase 3R-AB_4_-type alloy achieves a peak capacity of 345.6 mAh g^−1^ and retains 70.12% of its capacity after 200 cycles, outperforming the multiphase alloy annealed at 1070 °C, which has a capacity of 332.4 mAh g^−1^ and retains only 63.93% after 200 cycles. The appropriate annealing temperature helps to form a single and stable phase structure, avoiding the problem of unstable properties caused by the coexistence of multiple phases.

**Figure 7:**
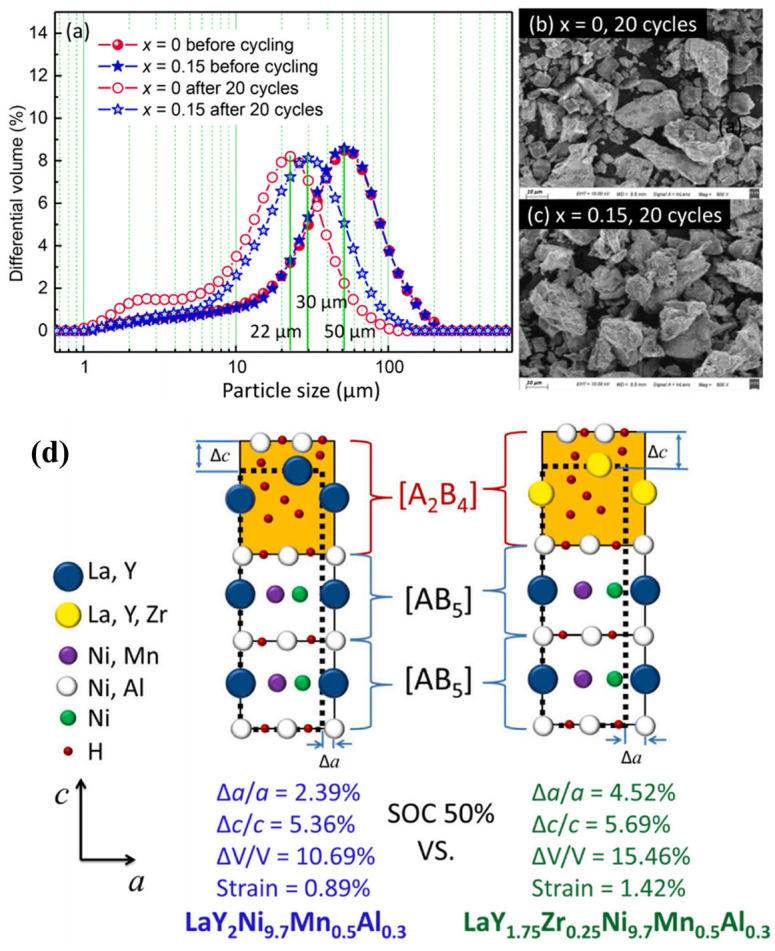
(**a**) Particle size distribution curves of the x = 0 and x = 0.15 alloys before cycling and after 20 cycles and SEM images of (**b**) x = 0 and (**c**) x = 0.15 alloy particles after 20 cycles; Reprinted with permission from Ref. [48]. Copyright 2021, copyright Liu, J et al. (**d**) the comparison of subunit structural deformation between the LaY_2_Ni_9.7_Mn_0.5_Al_0.3_ and LaY_1.75_Zr_0.25_Ni_9.7_Mn_0.5_Al_0.3_ alloys. Reprinted with permission from Ref. [82]. Copyright 2022, copyright Zhao, S et al.

The effect of elemental substitution in single-phase alloys can be directly attributed to structural changes (e.g., subunit volume matching) rather than interference from changes in phase composition. Synergistic or competitive effects between different phases are avoided in multiphase alloys. Liu et al. [48] synthesized La_0.75−x_Gd_x_Mg_0.25_Ni_3.5_ (x = 0, 0.05, 0.1, 0.15) alloys using induction melting, followed by annealing to achieve single-phase alloys. The XRD patterns of all alloys were consistent with a 2H-type (La, Mg)_2_Ni_7_ phase structure. Substituting Gd for La caused a reduction in both the lattice parameter and unit cell volume, as well as a decrease in the volume difference between the two subunits. In particular, when x = 0.15, the volumes of the two subunits became nearly identical, leading to a significant enhancement in the alloy’s cycling stability. Specifically, the capacity retention after 100 cycles increased from 82.1% to 88.2%. Among the single-phase alloys, the La_0.60_Gd_0.15_Mg_0.25_Ni_3.5_ alloy exhibits a well-matched subunit volume, resulting in a more synchronized hydrogen absorption and desorption process (Figure 7a–c). Zhao et al. [82] studied three groups of single-phase LaY_2_Ni_10.5_-based alloys, focusing on modifying the Ce_2_Ni_7_-type superlattice structure and hydrogen storage properties through Mn, Al, and Zr substitutions. Their findings revealed that replacing Ni with Mn and Al reduced the volume difference between the [A_2_B_4_] and [AB_5_] subunits, leading to a notable improvement in cycling stability. Specifically, the LaY_2_Ni_9.7_Mn_0.5_Al_0.3_ alloy achieved a maximum discharge capacity of 384.1 mAh g^−1^ with a capacity retention of 76.1% after 200 cycles. Although the substitution of Zr for Y resulted in zero subunit volume difference in LaY_1.75_Zr_0.25_Ni_9.7_Mn_0.5_Al_0.3_ alloy, the abnormal expansion of the a-axis (Δa/a = 4.52%) resulted in a lattice strain as high as 1.42% (Figure 7d), which in turn reduced the discharge capacity (367.4 mAh g^−1^) and cycle life (S_200_ = 60.9%). This indicates that the subunit structure deformation had a significant effect on the cycle life.

**Figure 8:**
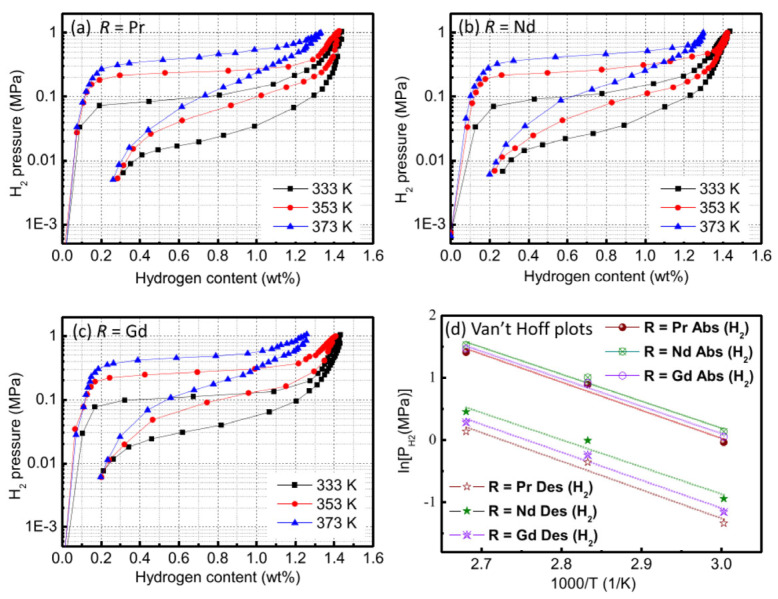
P-C-T curves of the alloys with (**a**) R = Pr, (**b**) R = Nd, and (**c**) R = Gd at 333, 353, and 373 K and (**d**) Van’t Hoff plots. Reprinted with permission from Ref. [84]. 2020, Liu, J et al.

Two hydrogen storage alloys with Ce_2_Ni_7_-type single-phase structures (LaNi_3.5_ and La_0.80_Mg_0.20_Ni_3.50_) were prepared by the stepwise sintering method by Cao et al. [83]. The introduction of Mg significantly increased the discharge capacity (364 mAh g^−1^). However, the particles were more susceptible to chalking (average particle size decreased from 58.2 μm to 17.4 μm) due to the volume expansion of the [A_2_B_4_] subunits and corrosion by Mg. This study emphasizes the need to balance capacity and cycling stability and to improve the overall performance of single-phase hydrogen storage alloys through structural optimization and anti-corrosion strategies. Liu et al. [84] investigated the hydrogen storage properties of A_2_B_7_-type single-phase La_0.60_R_0.15_Mg_0.25_Ni_3.45_ (R = Pr, Nd, Gd) alloys (Figure 8a–c) and found that Gd-substituted alloy retained 89.5% of the hydrogen storage capacity after 100 cycles, which was significantly superior to Pr (79.8%) and Nd (81.8%) substitutions. ΔH values for A_2_B_7_-type alloys range from −36 to −30 kJ mol^−1^ H_2_, with Gd-substituted alloys (e.g., La_0.60_Gd_0.15_Mg_0.25_Ni_3.45_) showing lower ΔH (−36.0 kJ mol^−1^) and higher plateau pressures (0.116 MPa). The smaller atomic radius of Gd (1.80 Å) preferentially replaces La in the [A_2_B_4_] subunit, reducing lattice microstrain (0.37%) and grain fragmentation (57.1 nm) during cycling, thus maintaining the stability of the hydrogen storage platform. The absolute values of ΔH decrease with a decreasing atomic radius (Pr > Nd > Gd), indicating that Gd alloys have the lowest hydride stability and the highest plateau pressure (Figure 8d). Lower ΔH alloys (e.g., Gd) have slower structural degradation during cycling due to less stress on hydride formation/decomposition and less microstrain accumulation. This suggests that single-phase [A_2_B_7_]-type alloys significantly enhance hydrogen storage cycling stability through structural homogeneity and subunit volume matching.

**Figure 9:**
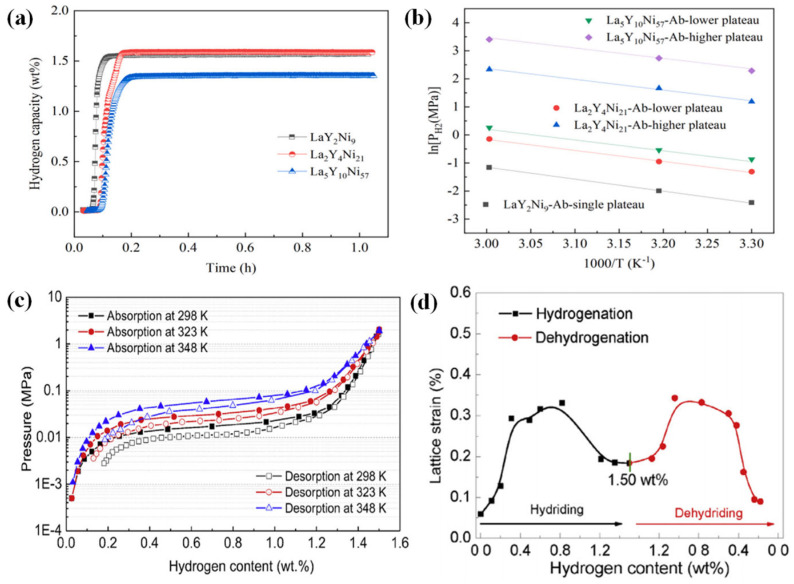
(**a**) First hydrogen absorption rate curve of La-Y-Ni-based single-phase alloys at 313 K; (**b**) Van’t Hoff plot of La-Y-Ni-based single-phase alloys; Adapted with permission from Ref. [62]. Copyright 2022, copyright He, X et al. (**c**) P-C isotherms for hydrogen absorption and desorption of La_0.78_Mg_0.22_Ni_3.67_Al_0.10_ compound; (**d**) the lattice strain of the compound during hydrogenation and dehydrogenation of the AB_4_-type La_0.78_Mg_0.22_Ni_3.67_Al_0.10_ compound. Adapted with permission from Ref. [85]. Copyright 2018, copyright Zhang, L et al.

Using the powder sintering method, He et al. [62] fabricated three La-Y-Ni-based hydrogen storage alloys: AB_3_-type LaY_2_Ni_9_, A_2_B_7_-type La_2_Y_4_Ni_21_, and A_5_B_19_-type La_5_Y_10_Ni_57_. Their findings indicate that the La_2_Y_4_Ni_21_ alloy exhibits the highest hydrogen storage capacity, which was 1.59 wt% at 313 K (Figure 9a). This is attributed to the fact that the A_2_B_7_-type structure contains more [A_2_B_4_] subunits, which have a larger crystal gap to accommodate more hydrogen atoms. The AB_3_ type has a slightly lower capacity (1.56 wt%) due to the high lattice strain, which makes it susceptible to amorphization. Increasing the [AB_5_]/[A_2_B_4_] ratio reduces the volume expansion during hydrogen uptake and release and enhances the cycling life, and La_5_Y_10_Ni_57_ has the best cycling stability (S_100_ = 49.50%) due to having the highest proportion of [AB_5_] subunits. With the increase in [AB_5_]/[A_2_B_4_] from 1:1 to 3:1, the absolute value of ΔH of the alloy decreases and the hydride stability decreases (Figure 9b). This research demonstrates that by adjusting the substituent ratio, a balance among hydrogen storage capacity, stability, and kinetic characteristics can be achieved, offering a crucial theoretical foundation for designing single-phase alloys.

Zhang et al. [85] synthesized a unique AB_4_-type single-phase superlattice alloy, La_0.78_Mg_0.22_Ni_3.67_Al_0.10_, through induction melting. This alloy boasts a high hydrogen storage capacity of 1.5 wt% and demonstrates exceptional cycling stability, retaining 90% of its capacity after 20 cycles (Figure 9c). AB_4_-type alloys (e.g., La_0.78_Mg_0.22_Ni_3.67_Al_0.10_) demonstrate near-ambient conditions for hydrogen release (ΔH = −23.2 kJ mol^−1^ for absorption, 24.2 kJ mol^−1^ for desorption). For La_0.78_Mg_0.22_Ni_3.67_Al_0.10_ (AB_4_ type), the activation energy for hydrogen desorption is ~24.2 kJ mol^−1^, facilitating room-temperature hydrogen release. By modulating the [A_2_B_4_]/[AB_5_] stacking ratio (1:4) and combining it with the selective occupation of Al atoms, they were able to reduce the volume difference between neighboring subunits from 8.24 Å^3^ to 3.02 Å^3^, with a lattice strain of only 0.3% (Figure 9d), significantly suppressing the structural deformation. The low enthalpy changes in hydrogen absorption/dehydrogenation (ΔH = −23.2/24.2 kJ mol^−1^) of this alloy provide excellent kinetics of hydrogen absorption/dehydrogenation at room temperature, which, combined with the anti-powdering ability of the superlattice structure, provides a new idea for hydrogen storage applications in a wide temperature range. Zhang et al. [86] designed an AB_4_-type single-phase hydrogen storage alloy, La_0.60_Sm_0.22_Mg_0.18_Ni_4.09_Al_0.09_Mn_0.10_, which has a maximum hydrogen storage capacity of 1.38 wt% at 298 K and retains 94% of its capacity after undergoing 50 cycles. The AB_4_-type alloy’s enthalpy change property (ΔH = −25.5 kJ mol^−1^ H_2_) renders it ideal for hydrogen storage and effective hydrogen release at room temperature, qualifying it as an excellent candidate for nickel–metal hydride batteries and hydrogen energy storage applications.

Table 6 shows a comparison of the properties of different single-phase superlattice hydrogen storage alloys. By investigating different types of single-phase alloys, a deeper insight into how phase structure affects hydrogen storage performance can be gained. In contrast to multiphase alloys, single-phase alloys minimize the interference from factors like phase interfaces. This allows researchers to examine more precisely how structural alterations impact the hydrogen storage performance of the alloys, thereby furnishing a more solid theoretical foundation for refining alloy design further.

In the research and application of hydrogen storage alloy materials, the performance characteristics of single-phase alloys and multiphase alloys show significant differences and complementarity. Single-phase alloys exhibit unique advantages due to their uniform crystal structure and chemical composition: Firstly, their structural simplicity provides an ideal model system for studying the structure-activity relationship between phase structure and hydrogen storage performance. For example, the La_0.60_Gd_0.15_Mg_0.25_Ni_3.5_ alloy achieves the optimization of the hydrogen atom diffusion path and the uniform distribution of lattice stress by constructing a single-phase Gd_2_Co_7_-type structure. This structural uniformity not only reduces the hindrance effect of the phase interface on hydrogen diffusion but can also effectively suppress the performance degradation caused by component segregation during the cycling process. In contrast, multiphase alloys can break through the performance limitations of single-phase materials through the synergistic effect between different phases. Taking the La_1.5_Y_1.5_Ni_12_ alloy as an example, the composite structure of its AB_4_ phase and the second phase forms a multi-level hydrogen storage platform. This multiphase system promotes differentiated responses of each component in terms of hydrogen adsorption enthalpy and lattice expansion coefficient and achieves the synergistic enhancement of hydrogen storage kinetics through interphase stress transfer and the interface hydrogen overflow effect. However, the stress concentration and element segregation that are prone to occur at the multiphase interface also led to the deterioration of its cycling stability, highlighting the inherent contradiction between structural complexity and stability.

Current research trends indicate that material selection needs to be systematically balanced based on the differentiated requirements of application scenarios. For application scenarios such as on-board hydrogen storage systems that require long-term stability, single-phase alloys have more advantages in defect passivation ability and structural integrity. In fixed hydrogen storage devices, the high-capacity characteristics of multiphase alloys can significantly increase the energy density of the system. It is worth noting that constructing multiphase structures through gradient design or optimizing multi-component components with the help of machine learning is becoming an important research direction to break through the performance bottleneck of single/multiphase systems, providing a new technical path for the development of next-generation intelligent hydrogen storage materials.

## 5. Effect of Rare-Earth Element Doping on the Phase Structure of Superlattice Hydrogen Storage Alloys

Research has demonstrated that doping/substituting the Y element significantly enhances the microstructure and properties of superlattice hydrogen storage alloys. Specifically, in La_0.65_Y_x_CaMgNi_9_ alloys (where x ranges from 0 to 0.15), Y doping modifies the phase composition of the alloy. An optimal amount of Y (x = 0.10) increases the abundance of the AB_5_ phase, narrows the volume difference between the [A_2_B_4_] and [AB_5_] subunits within the AB_3_ phase (decreasing ΔV from 3.106 Å^3^ to 1.665 Å^3^), and alleviates lattice strain during cycling. In terms of hydrogen storage properties, Y doping increases the hydrogen storage capacity and plateau pressure by increasing the hydrogen absorption sites and decreasing the hydride stability. The La_0.65_Y_x_CaMgNi_9_ alloy experiences an increase in hydrogen storage capacity from 1.73 to 1.93 wt%, with a plateau pressure of 0.29 MPa. The incorporation of Y reduces the enthalpy change of the hydride (from 30.2 kJ mol^−1^ to 19.9 kJ mol^−1^), facilitating hydrogen absorption and desorption at room temperature [67]. The smaller atomic radius of Y (0.178 nm) compared to La (0.187 nm) leads to a decrease in the unit cell volume upon substitution, enhancing the plateau pressure. In the La_1−x_Y_x_Mg_0.3_Ni_3.5_ alloy, when x = 0.35, the volume of the Gd_2_Co_7_-type phase crystal cell decreases from 0.788 nm^3^ to 0.765 nm^3^, resulting in an increase in the hydride decomposition pressure. Consequently, the desorption plateau pressure rises from 0.054 MPa to 0.356 MPa at 298 K [64].

**Figure 10:**
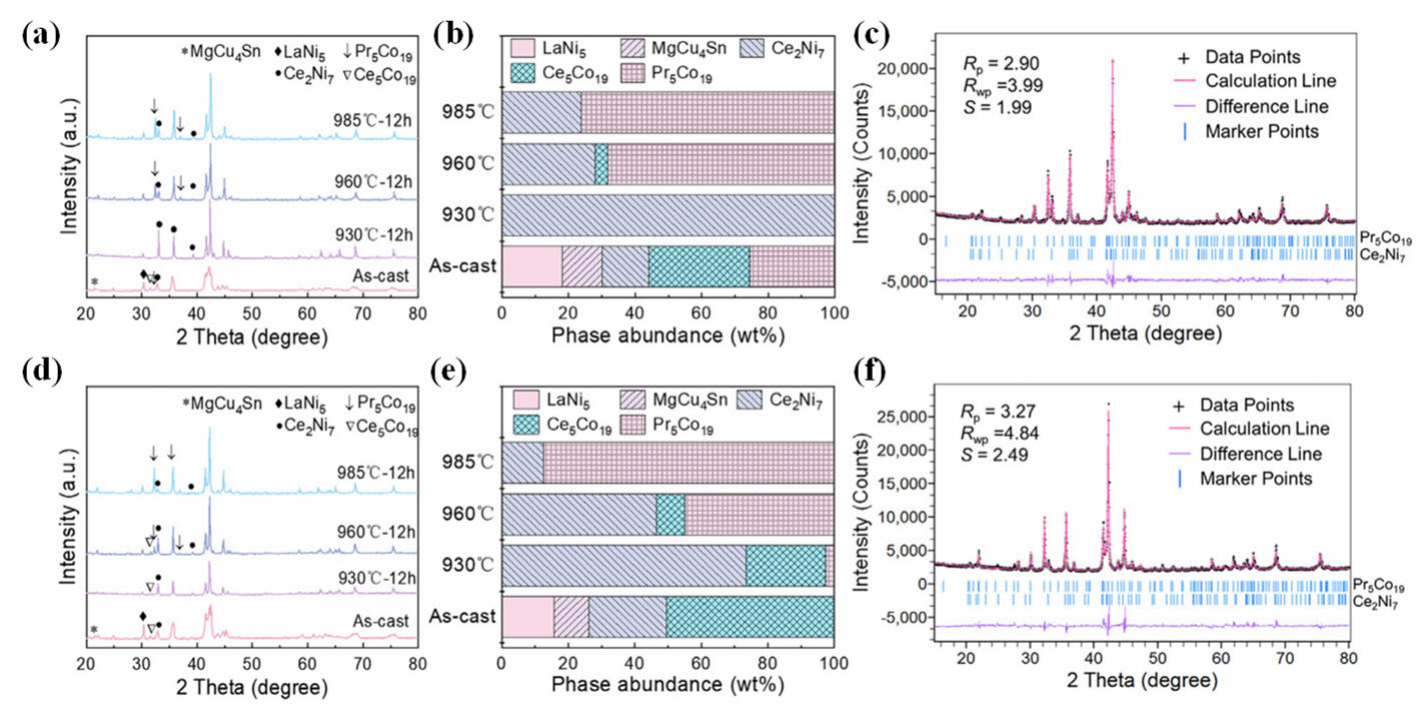
(**a**,**d**) XRD patterns and (**b**,**e**) phase composition of the as-cast alloys and annealed alloys at 930, 960, and 985 °C for La_0.62_Sm_0.15_Mg_0.23_Ni_3.55_Al_0.15_ and La_0.60_Sm_0.15_Y_0.02_Mg_0.23_Ni_3.55_Al_0.15_, respectively. Rietveld refinement results of the (**c**) La_0.64_Sm_0.16_Mg_0.20_Ni_3.56_Al_0.15_ and (**f**) La_0.62_Sm_0.16_Y_0.02_Mg_0.20_Ni_3.56_Al_0.15_ alloys annealed at 985 °C. Reprinted with permission from Ref. [68]. Copyright 2025, copyright Guo, Y et al.

Replacing La with Y in the A_5_B_19_-type La-Mg-Ni-based alloy significantly increases the proportion of the Pr_5_Co_19_-type phase to 76.2 wt% and prevents the Ce_2_Ni_7_ phase from becoming amorphous after annealing at 985 °C (Figure 10). Additionally, this substitution decreases the bulk mismatch coefficient from 1.41 to 1.29 and enhances capacity retention to 78.5%. The high electronegativity of Y (1.22 eV) reduces oxidative corrosion in alkaline environments and inhibits the formation of La(OH)_3_, which prolongs the cycling life [68]. In the La_0.96−x_Y_x_Mg_0.04_Ni_3.47−0.6x_Al_0.6x_ alloy, the combined substitution of Y and Al effectively optimizes the phase structure. Specifically, Y preferentially occupies the Ce_2_Ni_7_ phase, while Al enters the LaNi_5_ phase. This leads to a reduction in the Ce_2_Ni_7_ phase’s unit cell volume and an increase in the LaNi_5_ phase’s unit cell volume, minimizing the volume mismatch between the [A_2_B_4_] and [AB_5_] subunits. Consequently, the hydrogen storage capacity reaches 1.449 wt%, the hysteresis coefficient decreases from 0.407 to 0.302, and the reversibility of hydrogen adsorption/desorption thermodynamics is improved [87]. In conclusion, Y effectively enhances the overall performance of hydrogen storage alloys by adjusting the lattice structure via atomic substitution, refining the phase composition, and optimizing both thermodynamics and kinetics in a synergistic manner. Additionally, it also improves the corrosion and powdering resistance of the alloys. The impact of Y is closely related to the doping concentration, the alloy system, and the accompanying elements. Future research should delve deeper into the long-term stability of Y over a wide temperature range and its integration with innovative alloy designs.

Lim et al. discovered that the substitution of Ce for La in La_(0.65−x)_Ce_x_Ca_1.03_Mg_1.32_Ni_(9−y)_Al_y_ alloys significantly boosted the reversible hydrogen storage capacity to 71% when x = 0.5, albeit at a cost of reducing the maximum hydrogen storage capacity from 1.49 wt% to 1.04 wt%. Ce substitution for La promotes [AB_5_] phase formation and reduces [AB_3_] phase decomposition while boosting the platform pressure through lattice contraction at the expense of some capacity. Ce preferentially occupies the [AB_5_] subunits of the superlattice structure [88,89], inhibiting the generation of low-hydrogen-storage phases. In (LaCe)_x_Y_6−2x_Ni_18.5_Mn_1.5_Al (x = 0.95, 1.00, 1.15, 1.20, 1.25, 1.30) alloys, as the Ce content is increased from 0.95 to 1.30, the Ce_2_Ni_7_ phase share increases from 67.55 wt% to 90.84 wt%, suppressing the Gd_2_Co_7_ phase and increasing the plateau pressure [88].

In addition to modifying phase structure and plateau properties, rare-earth element doping can also refine an alloy’s microstructure, which plays a pivotal role in enhancing hydrogen storage kinetics. Existing studies have shown that microstructure refinement significantly increases the density of defects such as grain boundaries and dislocations, which act as fast diffusion channels for hydrogen atoms and thereby accelerate hydrogen absorption. For example, Zhang et al. [90] demonstrated that the mechanical milling of rare-earth Mg-Ni hydrogen storage alloys reduced the grain size to the nanoscale, resulting in a several-fold increase in hydrogen uptake rate. This enhancement arises from the abundant grain boundaries and increased surface area in the nanocrystalline structure, which provides more hydrogen adsorption sites and lowers the diffusion activation energy.

**Figure 11:**
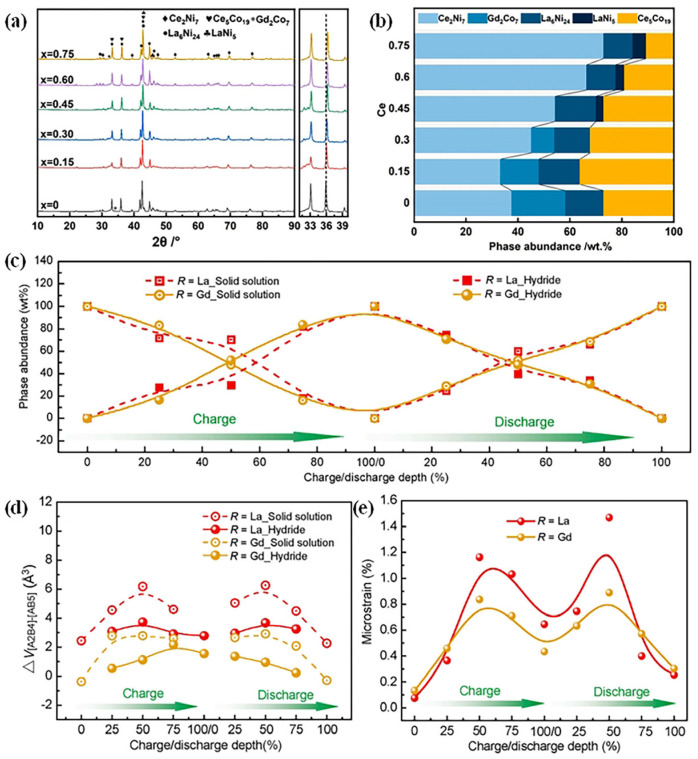
(**a**) XRD patterns of the La_1−x_Ce_x_Y_2_Ni_10.95_Mn_0.45_ alloys; (**b**) the change in phase abundance of the La_1−x_Ce_x_Y_2_Ni_10.95_Mn_0.45_ alloys with Ce content (Adapted with permission from Ref. [89]. Copyright 2024, copyright Liu, Y et al.) and (**c**) hydride phase abundances; (**d**) mismatch between [A_2_B_4_] and [AB_5_] subunits; and (**e**) microstrain of the alloys at different charge/discharge stages. Reprinted with permission from Ref. [94]. Copyright 2024, copyright Liu, J et al.

In the La_1−x_Ce_x_Y_2_Ni_10.95_Mn_0.45_ (x = 0, 0.15, 0.30, 0.45, 0.60, 0.75) alloys, an increase in Ce content led to an elevation in the Ce_2_Ni_7_ phase proportion, from 37.57 wt% to 72.94 wt% (Figure 11a,b). Specifically, at x = 0.45, the platform pressure rose from 0.04 MPa to 0.30 MPa, accompanied by a hydrogen storage capacity of 1.61 wt%. Notably, this alloy exhibited a single-platform property with remarkable stability, retaining 97.89% of its capacity after 100 cycles. The LaY_2_Ni_10.95_Mn_0.45_ alloy exhibits rapid hydrogen absorption, achieving 90% of its capacity within 5 min at 313 K, which is attributed to enhanced surface catalytic activity and hydrogen diffusion. Substitution with Ce in La_1−x_C_x_Y_2_Ni_10.95_Mn_0.45_ lowers the activation barrier, with ΔH decreasing from −32.66 kJ mol^−1^ (x = 0) to −27.38 kJ mol^−1^ (x = 0.75). Ce optimizes the pressure–capacity balance through lattice shrinkage and phase purification [89]. Although Ce substitution reduces the hydrogen storage capacity [65], a high hydrogen storage capacity can still be maintained through compositional optimization [89]. In the La_0.67_R_0.05_Y_0.13_Mg_0.15_Ni_3.70_Al_0.15_ alloy, substituting Ce for La boosts the high-rate discharge capability and enhances kinetics by improving surface catalytic activity and hydrogen diffusion (with a diffusion coefficient of 1.76 × 10^−1^ cm^2^ s^−1^) [91]. Ce plays a pivotal role in hydrogen storage alloys by balancing pressure, capacity, and cycle life through adjustments to phase structure, lattice contraction, and combinations of synergistic elements. This element holds considerable promise for applications, particularly in solid-state hydrogen storage.

Guo et al. [92] observed that substituting a portion of Y with Sm in La-Y-Ni-based alloys can prolong their cycling durability by bolstering both corrosion and pulverization resistance, despite a minor initial compromise in structural stability. Zhang et al. [93] revealed that Sm facilitates the emergence of Gd_2_Co_7_-type phases in La-Mg-Ni-based alloys, suppressing Ce_5_Co_19_-, CaCu_5_-, and MgCu_4_Sn-type phases, leading to substantial improvements in cycle stability (with a capacity retention of 87.7%) and a high-rate discharge capability of 40% at 1500 mAh g^−1^. In La-Y-Ni alloys, Sm enhances pulverization resistance by expanding the unit cell volume (specifically, increasing the c-axis from 24.347 Å to 24.368 Å) and fortifies surface corrosion resistance. In La-Mg-Ni-based alloys, Sm, due to its smaller atomic radius, promotes the formation of Gd_2_Co_7_-like phases (3R structure) and decreases the presence of non-A_2_B_7_ phases. This, in turn, reduces the development of internal strains and pulverization. Both investigations highlight the effectiveness of Sm in enhancing the overall functionality of hydrogen storage alloys.

Replacing La with Gd in La-Mg-Ni-based hydrogen storage alloys has been shown to improve both cycling stability and electrochemical properties. Liu et al. fabricated A_2_B_7_-type [48] and AB_3_-type [94] alloys through induction melting followed by annealing processes. Their research indicated that Gd primarily occupies the [A_2_B_4_] subunit, leading to a reduction in the volume difference between [A_2_B_4_] and [AB_5_] subunits and minimizing microstrain (Figure 11c–e). Consequently, the cycle stability of A_2_B_7_-type alloys improved from 82.1% to 88.2%, while for AB_3_-type alloys, it increased from 52% to 67.6%. Furthermore, the high-rate discharge capability at 1500 mAh g^−1^ also witnessed an elevation, from 48.2% to 52.4% for A_2_B_7_-type and from 53.6% to 66.4% for AB_3_-type alloys, which was attributed to the improved hydrogen diffusion kinetics. Additionally, the higher electronegativity of Gd (1.20 eV) compared to La (1.10 eV) acts as a barrier against oxidation reactions. In superlattice structures, hydrogen diffusion predominantly occurs via interstitial and grain boundary pathways. Interstitial diffusion involves hydrogen atoms hopping between lattice sites, governed by crystal symmetry and metal–hydrogen interactions. Grain boundary diffusion, on the other hand, occurs along disordered interfacial regions where atomic packing is irregular and defect concentration is high, resulting in lower diffusion activation energy. Zhao et al. [95] employed both theoretical calculations and experimental validation to demonstrate that in certain superlattice alloys, hydrogen interstitial diffusion exhibits low energy barriers, enabling rapid transport. In a separate study, Nowak et al. [60] employed mechanical alloying and annealing methods to investigate the effects of Gd incorporation. They found that an optimal Gd content (x = 0.25) boosts cycle stability, resulting in a capacity retention of 64.3% after 50 cycles. However, an excessive amount of Gd (x ≥ 0.5) resulted in a decline in capacity. These results suggest that the atomic radius and electronegativity advantages of Gd play crucial roles in optimizing the performance of these alloys. Collectively, the Gd modification strategy offers novel insights into the development of nickel–metal hydride battery anode materials that exhibit a long cycle life and high-power output.

In AB_3_-type hydrogen storage alloys, adding Pr helps to decrease the volume difference between the [A_2_B_4_] and [AB_5_] subunits, which in turn alleviates microstrain and the pulverization process. Specifically, in A_2_B_7_- and A_5_B_19_-type superlattice structures, Pr exhibits a preferential occupancy within the [AB_5_] subunit [96]. This leads to an augmentation in the content of the A_5_B_19_ phase, fostering the formation of a stable structure that effectively halts the decomposition of the A_2_B_7_ phase. Consequently, after undergoing 100 charge–discharge cycles, the capacity retention rate increases significantly from 85.1% to 90.7%. By strategically occupying lattice positions, Pr modifies the phase structure, creating a more stable subunit framework that efficiently halts pulverization, oxidation, and amorphization, ultimately prolonging cycle life and enhancing high-rate discharge performance. These advantages are especially evident in A_2_B_7_- and A_5_B_19_-type superlattice configurations, where an improvement in capacity retention of over 5% and an increase in high-rate discharge capability capability (HRD_X′_) of approximately 8% are noted.

Pulverization and corrosion are major degradation mechanisms limiting the long-term cycling stability of superlattice hydrogen storage alloys. Microstructural optimization and surface modification strategies have proven effective in mitigating these effects. Liu et al. [48] achieved a balanced sublattice volume between [A_2_B_4_] and [AB_5_] through selective atomic occupation while enhancing the alloy’s electronegativity. This design not only minimized volume mismatch and associated lattice strain but also improved oxidation and corrosion resistance.

Rare-earth elements, as the key components for optimizing the performance of hydrogen storage alloys, significantly enhance the comprehensive performance of the materials through a multi-dimensional collaborative mechanism: their unique atomic properties can regulate the lattice structure, expand the hydrogen storage gap, and reduce the volume expansion rate and precisely regulate the phase composition to construct a highly active hydrogen storage phase, achieving a simultaneous increase in capacity and kinetics. The thermodynamic and kinetic behaviors are optimized through electron orbital hybridization and surface active site design to reduce the hydrogen dissociation energy barrier and accelerate the diffusion rate. Meanwhile, by taking advantage of the passivation effect of rare-earth oxides and the buffering mechanism of the plastic phase, the corrosion resistance and anti-powdering ability are significantly enhanced, increasing the cycle life to more than 1.5 times that of conventional alloys. Current research focuses on rare-earth multi-element composite and interface engineering strategies, developing gradient structure materials with both a high capacity and long service life, providing more competitive solutions for new energy vehicles and renewable energy storage systems. Table 7 presents the effect of rare-earth modification on the hydrogen storage properties of superlattice hydrogen storage alloys. These discoveries offer crucial guidelines for developing innovative high-performance hydrogen storage materials.

## 6. Effect of Element Substitution on the Phase Structure of Superlattice Hydrogen Storage Alloys

Element substitution entails the incorporation of other elements into hydrogen storage materials, aimed at modulating the crystal structure, electronic configuration, physicochemical attributes, and hydrogen storage kinetics, thereby enhancing their hydrogen storage capabilities substantially. Research has demonstrated that elemental substitutions in La-Mg-Ni superlattice hydrogen storage alloys effectively optimize their structural configurations and mitigate inherent defects. This approach not only enhances the alloys’ overall hydrogen storage capacity but also significantly improves their stability. The underlying mechanisms of this element substitution strategy encompass several facets: (1) the element substitution can change the lattice structure of the alloy, thus affecting the atomic spacing, coordination number, and crystallization properties; (2) element substitution is capable of modifying the alloy’s electronic structure, thereby influencing both the electron state density and electron density distribution; (3) and element substitution can reduce the grain size of the alloy and improve its surface area and hydrogen storage kinetics. Table 4 shows the impact of element substitution on the hydrogen storage characteristics of superlattice hydrogen storage alloys.

**Figure 12:**
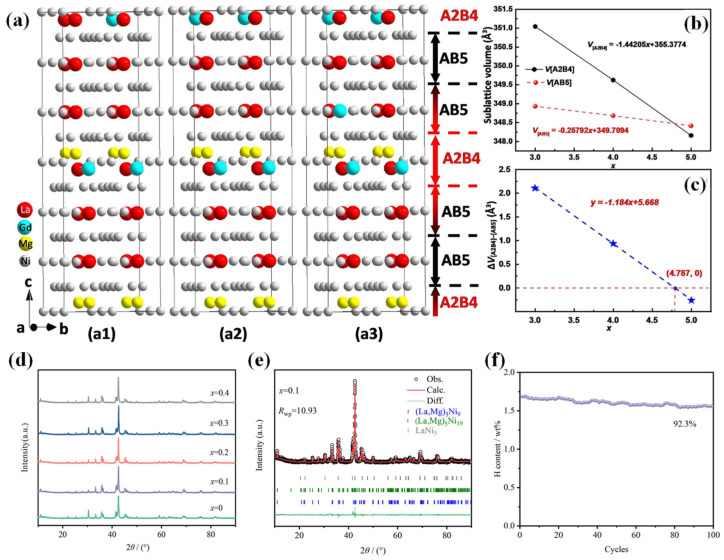
(**a**) Simulation models; (**b**) change trend of [A_2_B_4_] and [AB_5_] subunit volume; (**c**) subunit volume difference between [A_2_B_4_] and [AB_5_] with the increase in Gd content. Adapted with permission from Ref. [48]. Copyright 2021, copyright Liu, J et al. (**d**) XRD patterns of La_0.66_Mg_0.34_Ni_3.5–x_Co_x_; (**e**) Rietveld refinement and (**f**) cyclic property of the La_0.66_Mg_0.34_Ni_3.4_Co_0.1_ alloy. Adapted with permission from Ref. [97]. Copyright 2024, copyright He, X et al.

Based on the alloy structure analysis and first-principles calculation, Liu et al. [48] introduced an innovative strategy to balance the sublattice volumes of [A_2_B_4_] and [AB_5_] by selectively occupying Gd atoms, thereby improving the overall stability of the alloy (Figure 12a). As shown in Figure 12b, both [A_2_B_4_] and [AB_5_] sublattices shrink with the increase in Gd atomic proportion, and the volume difference between them is greatly reduced (Figure 12c), resulting in basically equal sublattice volumes of [A_2_B_4_] and [AB_5_] of La_0.6_Gd_0.15_Mg_0.25_Ni_3.5_. The results show that the alloy has stronger oxidation resistance and corrosion resistance than the original La_0.75_Mg_0.25_Ni_3.5_ alloy. La_0.6_Gd_0.15_Mg_0.25_Ni_3.5_ shows improved kinetics due to the reduced lattice strain between the A_2_B_4_ and AB_5_ subunits, with a hydrogen diffusion coefficient of ~1.2 × 10^−10^ cm^2^ s^−1^. He et al. [97] systematically investigated the influence of Co partial substitution for Ni on the hydrogen storage properties of the La_0.66_Mg_0.34_Ni_3.5−x_Co_x_ superlattice alloy system. XRD refining results (Figure 12d,e) show that the (La, Mg)_3_Ni_9_-phase content in the La_0.66_Mg_0.34_Ni_3.4_Co_0.1_ alloy increased significantly after Co substituted Ni. The hysteresis coefficient decreased significantly, and the reversible hydrogen storage capacity increased from 1.45 wt% to 1.60 wt%. La_0.66_Mg_0.34_Ni_3.4_Co_0.1_ exhibited reduced hysteresis (H_e_ = 0.76) compared to the undoped alloy (H_e_ = 1.04), which is linked to Co’s role in stabilizing subunit volumes. The capacity retention rate of La_0.66_Mg_0.34_Ni_3.4_Co_0.1_ alloy after Co substitution was 92.3% after 100 cycles, showing excellent cycle stability (Figure 12f). Moreover, Xu et al. [59] studied the hydrogen storage properties of A_2_B_7_-type (LaCe)_x_Y_6−2x_Ni_18.5_Mn_1.5_Al (x = 0.95, 1.00, 1.15, 1.20, 1.25, 1.30) alloys. When Y elements were replaced by La and Ce elements, the phase ratio of Ce_2_Ni_7_ increased from 67.55% to 90.84%, and the alloy showed excellent activation ability. After 100 cycles, the capacity retention rate of the alloy gradually increased from 63.2% (x = 0.95) to 69.2% (x = 1.30). Du et al. [64] used Y substitution strategy to adjust the hydrogen storage properties of La_0.7−x_Y_x_Mg_0.3_Ni_3.5_ alloy, and the substitution of Y element resulted in an increase in Gd_2_Co_7_ phase abundance. The hydrogen storage capacity of the La_0.42_Y_0.28_Mg_0.3_Ni_3.5_ alloy at 298 K was 1.55 wt%, and the La_0.42_Y_0.28_Mg_0.3_Ni_3.5_ alloy demonstrated remarkable cycling durability, with a capacity retention rate of 94.2% after 50 repeated hydrogen absorption/desorption cycles.

**Figure 13:**
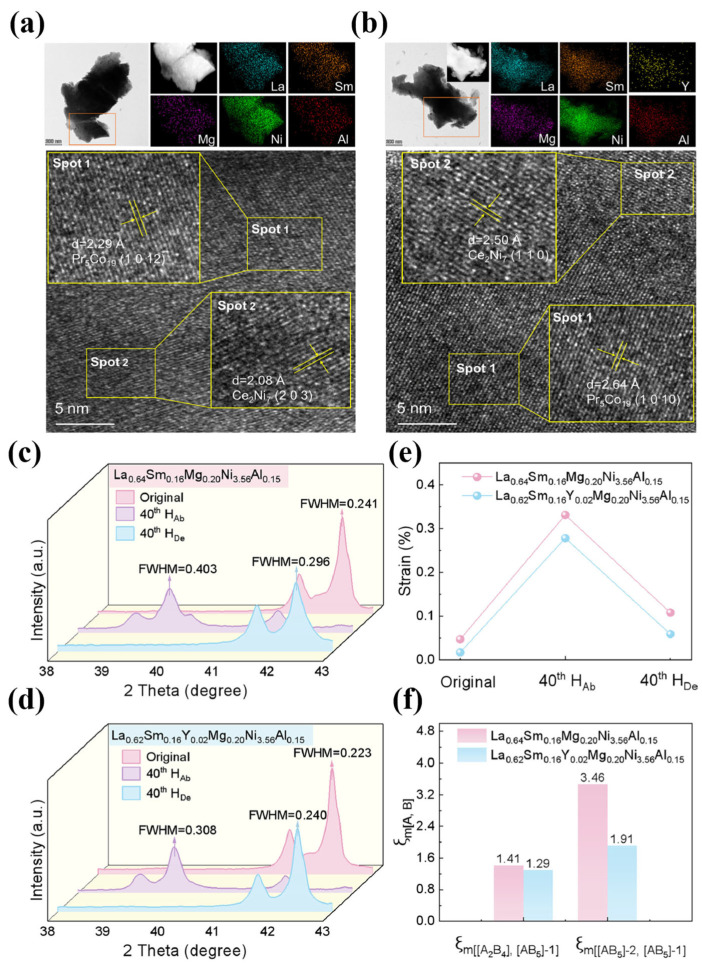
The TEM and HRTEM images of (**a**) La_0.64_Sm_0.16_Mg_0.20_Ni_3.56_Al_0.15_ and (**b**) La_0.62_Sm_0.16_Y_0.02_Mg_0.20_Ni_3.56_Al_0.15_ alloys; (**c**,**d**) FWHM change; (**e**) strain change, and (**f**) subunit volume mismatching coefficient of the La_0.62_Sm_0.16_Y_0.02_Mg_0.20_Ni_3.56_Al_0.15_ and La_0.64_Sm_0.16_Mg_0.20_Ni_3.56_Al_0.15_ alloys after 40 hydrogenation/dehydrogenation cycles. Adapted with permission from Ref. [68]. Copyright 2025, copyright Guo, Y et al.

Subsequently, Guo et al. [68] reduced the unit cell parameters of the alloy by partially replacing the La element with a Y element, promoted the formation of a Pr_5_Co_19_ phase, and improved the hydrogen storage performance of La Mg Ni-based alloy. HRTEM images (Figure 13a,b) show that La_0.64_Sm_0.16_Mg_0.20_Ni_3.56_Al_0.15_ and La_0.62_Sm_0.16_Y_0.02_Mg_0.20_Ni_3.56_Al_0.15_ alloys have two phases, Ce_2_Ni_7_ and Pr_5_Co_19_. Figure 13c indicates that after 40 hydrogenation and dehydrogenation cycles, La_0.64_Sm_0.16_Mg_0.20_Ni_3.56_Al_0.15_’s FWHM values are 0.241°, 0.403°, and 0.296°, respectively. However, the FWHM values of La_0.62_ Sm_0.16_Y_0.02_Mg_0.20_Ni_3.56_Al_0.15_ alloys are 0.223°, 0.308°, and 0.240°, which are all lower than those of the alloys without Y, indicating that the grain damage degree of the alloys with Y element is small. Meanwhile, lattice strain analysis further proves that the Y-element substitution alloy has better structural stability (Figure 13e). Additional research demonstrated that the substitution of the Y element serves to diminish the volumetric discrepancy between [A_2_B_4_] and [AB_5_] subunits. This substitution facilitates an improved volumetric compatibility among the subunits and contributes to the mitigation of lattice strain. Consequently, the alloy’s resistance to powdering is enhanced, leading to an overall augmentation in the cyclic stability of the material (Figure 13f). Simultaneously, the team prepared single-phase Pr_5_Co_19_-type alloys, namely La_0.58_Sm_0.18_Y_0.01_Mg_0.23_Ni_3.62_Al_0.16_ and La_0.58_Sm_0.21_Y_0.01_Mg_0.20_Ni_3.62_Al_0.16_. The research revealed that the incorporation of Mg can also effectively alleviate the lattice mismatch between [A_2_B_4_] and [AB_5_] subunits, reducing lattice strain during hydrogen absorption and desorption processes. Consequently, the modification leads to improved resistance against pulverization and enhanced structural integrity of the alloy [98]. Zhang et al. [99] proposed replacing Ni elements with low-cost Mn and Fe elements to achieve the purpose of cost reduction and increased efficiency. Research has revealed that after substituting Fe for Ni in the La_0.72_Y_0.13_Mg_0.15_Ni_3.65_Al_0.15_ alloy, the hydrogen storage capacity of the resultant La_0.72_Y_0.13_Mg_0.15_Ni_3.65_Al_0.15_Fe_0.05_ alloy increased from the original 1.23 wt% to 1.44 wt%, with the enthalpy change shifting from the initial −30.2 kJ mol^−1^ to −22.9 kJ mol^−1^. This indicates that the addition of Fe can improve the thermodynamic properties of the alloy, reducing the stability of the corresponding hydride to a certain extent.

**Figure 14:**
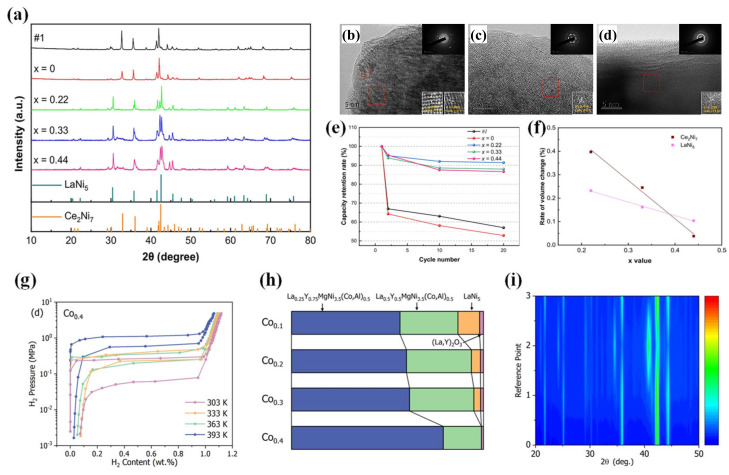
(**a**) XRD spectra; (**b**–**d**) HRTEM and SAED images after cycling; (**e**) capacity retention; (**f**) the relationship between the volume expansion rate of a unit cell and x of La_0.96−x_Y_x_Mg_0.04_Ni_3.47−0.6x_Al_0.6x_ superlattice alloys; Adapted with permission from Ref. [87]. Copyright 2024, copyright Wu, G et al. (**g**) P-C-T curves of the La_0.25_Y_0.75_MgNi_3.5_Co_0.4_Al_0.1_ alloy; (**h**) changes in phase composition and abundance of the La_0.25_Y_0.75_MgNi_3.5_Co_x_Al_0.5−x_ (x = 0.1, 0.2, 0.3, and 0.4) alloys; (**i**) XRD contour of La_0.25_Y_0.75_MgNi_3.5_Co_0.1_Al_0.4_ alloy. Adapted with permission from Ref. [100]. Copyright 2024, copyright Yin, W et al.

Previous studies have shown that the synergistic effect arising from the multi-element co-substitution strategy can better enhance the performance of alloys. Wu et al. [87] proposed a co-substitution strategy utilizing Y and Al and prepared a series of A_2_B_7_-type alloys, namely La_0.96_Mg_0.04_Ni_3.34_Al_0.13_ and La_0.96−x_Y_x_Mg_0.04_Ni_3.47−0.6x_Al_0.6x_ (x = 0, 0.22, 0.33, 0.44). Hysteresis coefficients (H_e_) are minimized in Y/Alco-doped alloys (e.g., La_0.52_Y_0.44_Mg_0.04_Ni_3.206_Al_0.264_, H_e_ = 0.302), reflecting improved reversibility. XRD spectral analysis revealed that the alloys consist of Ce_2_Ni_7_ and LaNi_5_ phases (Figure 14a). The unit cell volumes of Ce_2_Ni_7_ and LaNi_5_ phases exhibited contrasting trends with increasing x values: while the Ce_2_Ni_7_ phase demonstrated a contraction, the LaNi_5_ phase showed an expansion in unit cell volume. Notably, the synergistic substitution strategy employing Y and Al elements proved effective in significantly enhancing the hydrogen storage capacities of these alloys. When x = 0.4, the hydrogen storage capacity of the alloy at 303K increased from the original 0.903 wt% (x = 0) to 1.449 wt%, with a hysteresis coefficient of 0.302. The HRTEM and SAED images of the alloys after cycling further confirm the presence of Ce_2_Ni_7_ and LaNi_5_ phases within the alloys and reveal that there was no significant change in the alloy phase structure post-cycling (Figure 14b–d). The investigation further revealed that aluminum incorporation significantly improves the long-term hydrogen storage capacity retention rate of these alloys. This beneficial effect is primarily attributed to the effective suppression of lattice expansion by aluminum addition, which enhances the structural integrity and dimensional stability during hydrogen absorption/desorption cycles (Figure 14e,f). Then, Yin et al. [100] prepared a series of alloys with the composition La_0.25_Y_0.75_MgNi_3.5_Co_x_Al_0.5−x_ (x = 0.1, 0.2, 0.3, 0.4). The research demonstrated that Co doping effectively tailored the A_2_B_4_-type phase composition, whereas Al doping promoted the formation of the secondary LaNi_5_ phase. Specifically, the incorporation of Co elements expanded the interstitial lattice spacing, which in turn elevated the maximum hydrogen storage capacity of the alloys from the original 1.035 wt% to 1.118 wt% (Figure 14g). Conversely, the doping of Al elements reduced the hydrogen absorption/desorption platform pressure and hysteresis coefficient. As shown in Figure 14h, Co doping exerts a critical influence on both enhancing the compositional homogeneity of the reinforcing phase and increasing the phase fraction of the A_2_B_4_-type structure. Conversely, the doping of Al promotes the formation of the secondary phase (LaNi_5_). Investigations have demonstrated that upon hydrogen absorption, La-Mg-Ni_4_-based hydrogen storage alloys experience a remarkable structural transition from a cubic lattice to an orthorhombic framework. This transformation is manifested by the appearance of hydride diffraction peaks (Figure 14i). Meanwhile, Wang et al. [101] investigated the influence of co-substitution of Al and Y elements on the hydrogen storage properties of La-Mg-Ni alloys. The substitution of Ni atoms on the B-site with Al elements—which exhibit significantly larger atomic radii—effectively diminishes the atomic radius disparity between the A-site and B-site components, thereby mitigating the lattice distortion effects during the hydrogen absorption and desorption processes. The incorporation of Y preferentially replaces La in the [A_2_B_4_] structure, which can suppress the amorphous transformation hydrogen storage and release, enhancing the stability of the alloy.

In the component design and performance optimization of hydrogen storage alloys, the doping or substitution of different elements to regulate material properties presents complex synergistic or antagonistic effects, and its mechanism of action needs to be deeply analyzed from multiple scales. For example, in La-Mg-Ni-based alloys, the co-doping of rare-earth elements (such as Ce) and transition metals (such as Co) can produce a synergistic effect: Table 8 presents the effect of element substitution on the hydrogen storage properties of superlattice hydrogen storage alloys. The hybridization of the 4f electron orbitals of Ce and the 3d orbitals of Co reduces the hydrogen adsorption enthalpy by 18%, while the introduction of Co inhibits the surface oxidation of La elements. However, when Al partially replaces Ni, although the electron donor property of Al can increase the hydrogen diffusion coefficient, the lattice contraction it causes forms an antagonistic effect with the phase segregation phenomenon of Mg elements, resulting in a deterioration of cycling stability. This contradiction stems from the competitive relationship of doped elements in aspects such as electronic structure modulation, lattice distortion energy, and interface compatibility. The current research needs to combine first-principles calculations with in situ transmission electron microscopy technology to establish a quantitative correlation model among concentration-phase interfaces, especially for the cross-scale analysis of the dynamic evolution of long-range ordered structures in multivariate systems. Through high-throughput screening and machine learning-driven component design, it is expected to achieve a simultaneous breakthrough in hydrogen storage capacity and cycle life within the collaborative window, providing theoretical support for the development of the next generation of high-stability hydrogen storage materials.

## 7. Conclusions and Perspective

The comprehensive review concludes that preparation techniques, such as arc melting, induction melting, and powder metallurgy, are pivotal in the fabrication of superlattice hydrogen storage alloys with precise compositions. Furthermore, the incorporation of rare-earth elements through doping and elemental substitution serves as an effective strategy to tailor the phase structure of these alloys. The unique electronic configurations and large atomic radii of rare-earth elements facilitate lattice expansion, modification of the crystal structure symmetry, and promotion of specific phase formations, thereby influencing both the hydrogen storage capacity and the stability of hydride phases. Elemental substitution further modulates the lattice parameters, electron density distribution, and phase boundaries, potentially leading to the emergence of novel phases, elimination of undesired secondary phases, or refinement of microstructure. These alterations in phase structure profoundly impact the hydrogen absorption and desorption kinetics, as well as the cycling stability of the alloys. In essence, the strategic use of rare-earth doping and elemental substitution emerges as a crucial means to enhance the hydrogen storage and electrochemical properties of superlattice hydrogen storage alloys. Although remarkable achievements have been made in this field, several aspects remain to be explored:

(1) Further research is required to understand the combined impacts of multiple rare-earth element doping and multi-element substitution. The intricate interactions among these different elements may result in synergistic effects, potentially optimizing the phase structure and enhancing the overall properties of the alloys. This comprehensive understanding could lead to the design of alloys with superior hydrogen storage capabilities. As a type of hydrogen storage alloy material with excellent performance, the development of high-entropy superlattice hydrogen storage alloys will be a key research direction in the future.
(2) To gain deeper insights into the mechanisms governing the phase structure evolution during hydrogen absorption and desorption, in situ and operando characterization techniques should be more extensively employed. These advanced methodologies offer real-time monitoring, facilitating a clearer picture of the dynamic processes occurring within the alloys and aiding in the refinement of alloy design strategies.
(3) Considering the practical application of hydrogen storage alloys, emphasis must be placed on cost-effective material design and scalable production processes. Balancing performance and cost is paramount to the widespread adoption of these alloys in the hydrogen economy. Innovations in material science and manufacturing techniques are essential to ensure that high-performance superlattice hydrogen storage alloys can be produced economically and in large quantities, contributing to a sustainable energy future.
(4) Building upon advancements in material design and scalable synthesis, future efforts should prioritize the translation of superlattice hydrogen storage alloys into functional energy storage systems, particularly nickel–metal hydride (Ni-MH) batteries and high-pressure hydrogen storage tanks. These alloys exhibit favorable hydrogen absorption kinetics and tunable electrochemical properties, making them promising candidates for next-generation energy storage solutions. However, their commercial implementation remains constrained by unresolved challenges, including integration compatibility, operational reliability under real-world conditions, and the absence of unified engineering standards for system-level deployment. To bridge the gap between laboratory innovation and industrial application, a concerted interdisciplinary approach is required—one that combines advanced alloy development, device-level optimization, and techno-economic analysis. Establishing robust pathways from material discovery to market readiness will be essential for unlocking the full potential of superlattice hydrogen storage alloys in the future hydrogen economy.
(5) Significant advancements have been achieved in the development of superlattice hydrogen storage alloys; however, several critical challenges must be addressed to facilitate their practical application and large-scale deployment.
  i. A primary obstacle lies in the high material costs and complexity of manufacturing processes, notably the reliance on high-purity rare-earth elements and energy-intensive synthesis methods such as arc melting. Developing cost-effective material design strategies alongside scalable manufacturing technologies is essential to produce high-performance alloys more economically. Additionally, controlling interfacial defects, hydrogen embrittlement, and corrosion phenomena during large-scale fabrication remains a significant hurdle, as these factors increase production costs and compromise the long-term stability of the alloys. Lattice distortion and phase separation occurring during repeated hydrogen absorption and desorption cycles further contribute to capacity fade and impede commercialization efforts, primarily due to insufficient long-term thermodynamic stability.
  ii. Advanced in situ and operando characterization techniques, including X-ray diffraction (XRD) and transmission electron microscopy (TEM), provide critical insights into the dynamic structural evolutions within these alloys under operational conditions. These tools enable real-time monitoring of processes such as hydrogen-induced crack propagation, thereby informing strategies to optimize alloy design and improve performance. Furthermore, machine learning (ML) methodologies, such as generative adversarial networks (GANs), have been employed to expedite the optimization of rare-earth element ratios, reducing development timelines and enhancing resistance to hydrogen embrittlement. By elucidating quantitative relationships among composition, processing parameters, and electrochemical performance, ML approaches serve as powerful accelerators for discovering novel alloy architectures and refining material design.
  iii. To overcome remaining barriers to large-scale implementation, future research should prioritize the development of cost-effective and scalable synthesis protocols. This includes exploring alternative alloy compositions with reduced rare-earth content or incorporating transition metals to enhance resource sustainability. Long-term durability studies at the atomic level, utilizing computational thermodynamics and defect engineering strategies, are urgently needed to understand and mitigate degradation mechanisms. Additionally, hybrid systems that integrate superlattice alloys with porous scaffolds or 2D materials, such as MXenes and graphene, offer promising avenues to enhance kinetic properties and structural stability. Incorporating sustainability considerations, such as life-cycle analysis, into materials development frameworks will ensure alignment with global decarbonization objectives. Through these combined efforts—advancing materials science, innovative characterization methods, and sustainable design principles—next-generation superlattice hydrogen storage alloys with superior performance, economic viability, and durability can be realized, supporting the transition towards sustainable energy systems.

## Figures and Tables

**Table 1:** Comparison of hydrogen storage properties between superlattice alloys and other metal hydrides.

Property	Superlattice Alloys	Mg-Based Hydrides	Complex Hydrides
Theoretical Hydrogen Storage Capacity	<2 wt%	<7.6 wt%	<18 wt% (LiBH_4_)
Operating Temperature	25–80 °C	250–400 °C (catalyst required)	100–400 °C (dependent components)
Hydrogen Absorption/Desorption Kinetics	Quicker (nanomaterial structure optimization)	Slow (need ball milling/doping for improvement)	Very slow (requires nano-scale confinement or catalysts)
Key Limitation	High synthesis cost	Sluggish dehydrogenation kinetics	Poor reversibility

**Table 2:** Summary of phase structure of superlattice hydrogen storage alloys obtained by induction melting method.

Preparation Method	Alloy Ratio	Annealing/ Sintering Temperature	Phases	Abundance (wt%)	Hydrogen Storage Capacity (wt%)	Cyclic Stability	Ref.
Induction Melting + Annealing	La_0.60_Pr_0.15_Mg_0.25_Ni_3.25_	1253 K	2H-(La, Mg)_2_Ni_7_	100	/	/	[63]
	La_0.60_Sm_0.20_Mg_0.20_Ni_3.50_Al_0.20_	1243 K	A_5_B_19_	/	/	88.7% (100 cycles)	[66]
			CaCu_5_	/			
			AB_4_	62.5			
	La_0.65_Ca_1.03_Mg_1.32_Ni_9_	/	A_3_B_9_	78	1.60	/	[65]
			AB_5_	22			
	La_0.15_Ce_0.5_Ca_1.03_Mg_1.32_Ni_6.5_Al_2.5_		A_3_B_9_	7	0.70	/	
			AB_5_	29			
			AB_2_	18			
			A_2_B_7_	5			
	La_0.65_CaMgNi_9_	1223 K	A_3_B_9_	79.6	1.73	87% (100 cycles)	[67]
			AB_5_	20.4			
	La_0.65_Y_0.05_CaMgNi_9_		A_3_B_9_	59.0	1.80	/	
			AB_5_	41.0			
	La_0.65_Y_0.10_CaMgNi_9_		A_3_B_9_	54.6	1.97	91% (100 cycles)	
			AB_5_	45.4			
	La_0.65_Y_0.15_CaMgNi_9_		A_3_B_9_	49.1	1.54	/	
			AB_5_	50.9			
	La_0.75−x_Gd_x_Mg_0.25_Ni_3.5_ (x = 0.15)	1223 K, 1263 K, 1273 K, 1283 K	2H-type (La,Mg)_2_Ni_7_	100	/	88.2% (100 cycles)	[48]
	La_0.7_Mg_0.3_Ni_3.5_	/	Gd_2_Co_7_	6.9	1.63	/	[64]
			PuNi_3_	75.8			
			CaCu_5_	17.3			
	La_0.56_Y_0.14_Mg_0.3_Ni_3.5_		Gd_2_Co_7_	24.8	1.59	/	
			PuNi_3_	61.1			
			CaCu_5_	14.1			
	La_0.42_Y_0.28_Mg_0.3_Ni_3.5_		Gd_2_Co_7_	36.9	1.55	94.2% (50 cycles)	
			PuNi_3_	46.6			
			CaCu_5_	16.5			
	La_0.35_Y_0.35_Mg_0.3_Ni_3.5_		Gd_2_Co_7_	52.9	1.43	/	
			PuNi_3_	38.4			
			CaCu_5_	8.7			
	La_0.62_Sm_0.15_Mg_0.23_Ni_3.55_Al_0.15_	1203 K	Ce_2_Ni_7_	100	/	/	[68]
	La_0.60_Sm_0.15_Y_0.02_Mg_0.23_Ni_3.55_Al_0.15_		Ce_2_Ni_7_	73.4	1.32	93% (40 cycles)	
			Ce_5_Co_19_	23.9			
			Pr_5_Co_19_	2.7			
		1148 K	2H-A_2_B_7_	60.16	/	/	
			3R-A_2_B_7_	39.84			
		1323 K	2H-A_2_B_7_	100	/	/	
		1398 K	2H-A_2_B7	61.64	/	/	
			2H-A_5_B_19_	36.74			
			Y_2_O_3_	1.62			
	La_0.60_Sm_0.15_Y_0.02_Mg_0.23_Ni_3.55_Al_0.15_		Ce_2_Ni_7_	73.4	1.28	89% (40 cycles)	
			Ce_5_Co_19_	23.9			
			Pr5Co19	2.7			
		1148 K	2H-A_2_B_7_	60.16	/	/	
			3R-A_2_B_7_	39.84			
		1323 K	2H-A_2_B_7_	100	/	/	
		1398 K	2H-A_2_B_7_	61.64	/	/	
			2H-A_5_B_19_	36.74			
			Y_2_O_3_	1.62			

**Table 3:** Summary of phase structure of superlattice hydrogen storage alloys obtained by arc melting method.

Preparation Method	Alloy Ratio	Annealing/ Sintering Temperature	Phases	Abundance (wt%)	Hydrogen Storage Capacity (wt%)	Cyclic Stability	Ref.
Arc Melting + Annealing	Pr_5_Ni_19_	1403 K	Sm_5_Co_19_	100	1.1	/	[69]
	Nd_2_Ni_7_	1448 K	Ce_2_Ni_7_	88	1.22	/	[62]
			CaCu_5_	12			
	GdNi_3_	1223 K	PuNi_3_	98	1.07	/	[70]
			CaCu_5_	2			
	LaY_2_Ni_10.5_	/	La_7_Ni_16_	1.0	1.49	/	[72]
			PuNi_3_	11.6			
			Ce_2_Ni_7_	21.9			
			Gd_2_Co_7_	38.0			
			Ce_5_Co_19_	15.7			
			CaCu_5_	11.8			
		1273 K	Gd_2_Co_7_	100	/	/	
		1373 K	Ce_2_Ni_7_	100	/	/	
	La_1.5_Y_1.5_Ni_12_	1373 K	3R-AB_4_	50.9	1.33	/	[73]
			AB_5_	4.92			
			2H-Pr_5_Co_19_	44.1			
		1388 K	3R-AB_4_	62.2			
			2H-Pr_5_Co_19_	37.7			
		1393 K	3R-AB_4_	100			
		1398 K	3R-AB_4_	93.6			
			AB_5_	6.32			
	La_1.5_Y_1.5_Ni_11_Mn_1.0_	1323 K	2H-Pr_5_Co_19_	100	/	/	
		1343 K	3R-AB_4_	68.3			
			AB_5_	18.9			
			2H-Pr_5_Co_19_	12.6			
		1358 K	3R-AB_4_	94.1			
			2H-Pr_5_Co_19_	5.86			
		1361 K	3R-AB_4_	100			
		1368 K	3R-AB_4_	75.2			
			AB_5_	24.8			
	(La_0.33_Y_0.67_)_5_Ni_18.1_Mn_0.9_	1423 K	2H-Pr_5_Co_19_	95.8	/	/	[74]
			2H-Ce_2_Ni_7_	4.2			
	(La_0.33_Y_0.67_)5Ni_17.8_Mn_0.9_Al_0.3_	1373 K	2H-Pr_5_Co_19_	88.8	/	/	
			2H-Ce_2_Ni_7_	11.2			
	(La_0.33_Y_0.67_)5Ni_17.5_Mn_0.9_Al_0.6_		2H-Pr_5_Co_19_	85.8	/	69.8 (200 cycles)	
			2H-Ce_2_Ni_7_	14.2			
	(La_0.33_Y_0.67_)5Ni_17.2_Mn_0.9_Al_0.9_		2H-Pr5Co_19_	93.4	/	/	
			2H-Ce_2_Ni_7_	6.6			
		1148 K	2H-A_2_B_7_	60.16	/	/	
			3R-A_2_B_7_	39.84			
		1323 K	2H-A_2_B_7_	100			
		1398 K	2H-A_2_B_7_	61.64			
			2H-A_5_B_19_	36.74			
			Y_2_O_3_	1.62			

**Table 4:** Summary of phase structure of superlattice hydrogen storage alloys obtained by sintering method.

Preparation Method	Alloy Ratio	Annealing/ Sintering Temperature	Phases	Abundance (wt%)	Hydrogen Storage Capacity (wt%)	Cyclic Stability	Ref.
High-Pressure Sintering	La_0.25_Mg_0.75_Ni_3.5_- 1.5 GPa	1173 K	Ce_2_Ni_7_	35.9	/	/	[76]
			Pr_5_Co_19_	64.1			
	La_0.25_Mg_0.75_Ni_3.5_- 2.0 GPa		Ce_2_Ni_7_	45.1	/	/	
			Pr_5_Co_19_	53.0			
			LaNi_5_	1.9			
	La_0.25_Mg_0.75_Ni_3.5_- 2.5 GPa		Ce_2_Ni_7_	53.2	/	/	
			Pr_5_Co_19_	42.0			
			LaNi_5_	4.8			
	La_0.25_Mg_0.75_Ni_3.5_- 4.0 GPa		Ce_2_Ni_7_	49.4	/	/	
			Pr_5_Co_19_	31.0			
			LaNi_5_	22.6			
			MgNi_2_	6.2			
Thermal Diffusion Sintering Method	Mg(0)/La_0.74_Sm_0.03_Y_0.23_Ni_4.32_Al_0.04_ (1)	1023 K	LaNi_5_	66	1.48	/	[77]
			Gd_2_Co_7_	12			
			Ce_5_Co_19_	22			
	Mg(0.17)/La_0.74_Sm_0.03_Y_0.23_Ni_4.32_Al_0.04_ (0.83)		Gd_2_Co_7_	46	/	/	
			Ce_5_Co_19_	54			
	Mg(0.21)/La_0.74_Sm_0.03_Y_0.23_Ni_4.32_Al_0.04_ (0.79)		Gd_2_Co_7_	44	/	/	
			Ce_2_Ni_7_	56			
	Mg(0.28)/La_0.74_Sm_0.03_Y_0.23_Ni_4.32_Al_0.04_ (0.72)		Gd_2_Co_7_	64	1.72	89.33% (20 cycles)	
			Ce_2_Ni_7_	36			
	Mg(0.33)/La_0.74_Sm_0.03_Y_0.23_Ni_4.32_Al_0.04_ (0.67)		MgCu_4_Sn	4	/	/	
			Gd_2_Co_7_	70			
			Ce_2_Ni_7_	26			
	Mg(0.38)/La_0.74_Sm_0.03_Y_0.23_Ni_4.32_Al_0.04_ (0.62)		MgCu_4_Sn	14	1.69	/	
			Gd_2_Co_7_	59			
			Ce_2_Ni_7_	27			
Stepwise Sintering Method	La_0.6_Gd_0.15_Mg_0.25_Ni_3.8_	1203 K	Pr_5_Co_19_	100	/	/	[75]
	La_0.6_Gd_0.15_Mg_0.25_Ni_3.5_		Ce_2_Ni_7_	100			
	La_0.6_Gd_0.15_Mg_0.25_Ni_3.0_		PuNi_3_	100			
	La_2_MgNi_9_	1293 K	3R-AB_3_	100	1.596	/	[56]
	La_3_MgNi_14_		2H-A_2_B_7_	100	1.587	/	
	La_4_MgNi_19_		2H,3R-A_5_B_19_	/	1.569	/	

**Table 5:** Summary of phase structure of superlattice hydrogen storage alloys obtained by other methods.

Preparation Method	Alloy Ratio	Annealing/ Sintering Temperature	Phases	Abundance (wt%)	Hydrogen Storage Capacity (wt%)	Cyclic Stability	Ref.
Induction Melting + Annealing	La_2_Y_4_Ni_20.8_Mn_1.2_Al_0.8_	1148 K	Ce_2_Ni_7_	52.36	1.48	78.9 (100 cycles)	[61]
			GdCo_5_	15.61			
			Ce_5_Co_19_	32.03			
Rapid Quenching + Annealing			Ce_2_Ni_7_	59.58	1.52	87.5 (100 cycles)	
			Gd_2_Co_7_	40.42			
Gas Atomization + Annealing			Ce_2_Ni_7_	50.21	1.40	90.5 (100 cycles)	
			Gd_2_Co_7_	35.72			
			GdCo_5_	14.08			
Levitation Melting + Annealing	La_0.85_Mg_0.15_Ni_2.65_Co_1.05_Zr_0.1_	1173 K	LaNi_5_	52.05	/	88.3 (100 cycles)	[80]
			Ce_5_Co_19_	17.29			
			Pr_5_Co_19_	10.35			
			Ce_2_Ni_7_	20.31			
	La_0.85_Mg_0.15_Ni_2.65_Co_1.05_Cr_0.1_		LaNi_5_	41.12	/	77.9 (100 cycles)	
			Ce_5_Co_19_	31.88			
			Pr_5_Co_19_	24.24			
			Ce_2_Ni_7_	2.76			
	La_0.85_Mg_0.15_Ni_2.65_Co_1.05_Al_0.1_		LaNi_5_	44.82	/	86.7 (100 cycles)	
			Ce_5_Co_19_	28.68			
			Pr_5_Co_19_	8.26			
			Ce_2_Ni_7_	18.24			
	La_0.85_Mg_0.15_Ni_2.65_Co_1.05_Mn_0.1_		LaNi_5_	37.69	/	75.8 (100 cycles)	
			Ce_5_Co_19_	33.38			
			Pr5Co_19_	20.96			
			Ce_2_Ni_7_	7.98			
	La_0.85_Mg_0.15_Ni_2.65_Co_1.05_Ni_0.1_		LaNi_5_	6.80	/	82.4 (100 cycles)	
			Ce_5_Co_19_	83.60			
			Pr_5_Co_19_	9.61			
Rapid Quenching + Annealing	LaY_1.9_Ni_10_Mn_0.5_Al_0.2_	/	2H-A_2_B_7_	45.40	/	/	[55]
			3R-A_2_B_7_	30.50			
			3R-A_5_B_19_	18.08			
			AB_5_	6.02			
		1148 K	2H-A_2_B_7_	60.16	/	/	
			3R-A_2_B_7_	39.84			
		1323 K	2H-A_2_B_7_	100	/	/	
		1398 K	2H-A_2_B_7_	61.64	/	/	
			2H-A_5_B_19_	36.74			
			Y_2_O_3_	1.62			

**Table 6:** Comparison of properties of different single-phase superlattice hydrogen storage alloys.

Alloy	Hydrogen Absorption Capacity/wt%	Reversible Capacity/wt%	Platform Pressure/MPa		ΔH/ kJ mol^−1^		Ref.
			Absorption	Desorption	Absorption	Desorption	
LaY_2_Ni_10.5_	1.49	1.29	0.26	/	/		[72]
La_1.5_Y_1.5_Ni_12_	1.33	0.8	5.8	0.05	/		[73]
La_0.60_Pr_0.15_Mg_0.25_Ni_3.45_	1.44	1.13	0.097	0.027	−37.5	38.2	[84]
La_0.60_Nd_0.15_Mg_0.25_Ni_3.45_	1.43	1.16	0.106	0.039	−36.6	37.3	
La_0.60_Gd_0.15_Mg_0.25_Ni_3.45_	1.43	1.22	0.116	0.116	−36.0	36.2	
LaY_2_Ni_9_	1.56	/	/		−35.23	/	[62]
La_2_Y_4_Ni_21_	1.59	/	/		−32.86	/	
La_5_Y_10_Ni_57_	1.35	/	/		−31.92	/	
La_0.78_Mg_0.22_Ni_3.67_Al_0.10_	1.50	/	/		−23.2	24.2	[85]
La_0.60_Sm_0.22_Mg_0.18_Ni_4.09_Al_0.09_Mn_0.10_	1.38	/	0.029	/	−25.5	28.3	[86]

**Table 7:** Comparison of properties of different rare-earth-element-modified superlattice hydrogen storage alloys.

Alloy	Hydrogen Absorption Capacity/wt%	Reversible Capacity/wt%	Platform Pressure/MPa		H_f_	ΔH/kJ mol^−1^		Ref.
			Absorption	Desorption		Absorption	Desorption	
La_0.65_Y_0.10_CaMgNi_9_	1.93	1.91	0.29	/		−27.1	26.8	[67]
La_0.52_Y_0.44_Mg_0.04_Ni_3.21_Al_0.26_	1.449	/	0.188	0.139	0.302	−29.216	27.572	[87]
La_0.7_Mg_0.3_Ni_3.5_	1.63	1.42	0.088	0.054	0.49	/		[64]
La_0.56_Y_0.14_Mg_0.3_Ni_3.5_	1.59	1.51	0.154	0.088	0.56	/		
La_0.42_Y_0.28_Mg_0.3_Ni_3.5_	1.55	1.49	0.489	0.244	0.70	−32.18	31.01	
La_0.35_Y_0.35_Mg_0.3_Ni_3.5_	1.43	1.39	0.617	0.356	0.55	/		
La_0.64_Sm_0.15_Mg_0.20_Ni_3.56_Al_0.15_	1.28	/	0.019	/		−33.9	35.3	[68]
La_0.62_Sm_0.16_Y_0.02_Mg_0.20_Ni_3.56_Al_0.15_	1.32	/	0.022	/		−32.3	34.6	
La_0.65_Ca_1.03_Mg_1.32_Ni_9_	1.49	1.16	0.10	0.04		/		[65]
La_1_Y_2_Ni_10.95_Mn_0.45_	1.64	0.56	0.14/0.04	0.09/0.03	0.41/0.43	−32.66/−33.87	/	[89]
La_0.85_Ce_0.15_Y_2_Ni_10.95_Mn_0.45_	1.65	1.06	0.18/0.07	0.15/0.05	0.22/0.44	−32.06/−31.96	/	
La_0.70_Ce_0.30_Y_2_Ni_10.95_Mn_0.45_	1.62	1.20	0.27/0.11	0.22/0.07	0.18/0.41	−31.60/−30.75	/	
La_0.55_Ce_0.45_Y_2_Ni_10.95_Mn_0.45_	1.61	1.35	0.30	0.23	0.26	−29.95	/	
La_0.40_Ce_0.60_Y_2_Ni_10.95_Mn_0.45_	1.57	1.23	0.36	0.25	0.37	−28.25	/	
La_0.25_Ce_0.75_Y_2_Ni_10.95_Mn_0.45_	1.56	1.08	0.58	0.33	0.56	−27.38	/	
La_0.67_R_0.05_Y_0.13_Mg_0.15_Ni_3.70_Al_0.15_	1.23	/	0.008	0.004	0.69	−31.2	34.3	[91]
La_0.67_R_0.05_Y_0.13_Mg_0.15_Ni_3.70_Al_0.15_	1.46	/	0.010	0.004	0.92	−38.1	46.3	
La_0.67_R_0.05_Y_0.13_Mg_0.15_Ni_3.70_Al_0.15_	1.50	/	0.015	0.008	0.63	−30.3	26.6	
La_0.67_R_0.05_Y_0.13_Mg_0.15_Ni_3.70_Al_0.15_	1.43	/	0.014	0.003	1.54	−30.3	26.6	
La_0.67_R_0.05_Y_0.13_Mg_0.15_Ni_3.70_Al_0.15_	1.37	/	0.011	0.007	0.45	−34.0	31.1	

**Table 8:** Comparison of properties of modified superlattice hydrogen storage alloys replaced by different elements.

Alloy	Hydrogen Absorption Capacity/ wt%	Reversible Capacity/wt%	Platform Pressure/MPa		H_f_	ΔH/kJ mol^−1^		Ref.
			Absorption	Desorption		Absorption	Desorption	
La_0.66_Mg_0.34_Ni_3.5_	1.63	1.45	0.169	0.060	1.04		30.84	[97]
La_0.66_Mg_0.34_Ni_3.4_Co_0.1_	1.77	1.60	0.131	0.061	0.76	/	29.85	
La_0.66_Mg_0.34_Ni_3.3_Co_0.2_	1.69	1.53	0.128	0.062	0.72		29.18	
La_0.66_Mg_0.34_Ni_3.2_Co_0.3_	1.73	1.55	0.124	0.065	0.64		27.10	
La_0.66_Mg_0.34_Ni_3.1_Co_0.4_	1.70	1.59	0.132	0.079	0.51		26.02	
La_0.7_Mg_0.3_Ni_3.5_	1.63	1.42	0.088	0.054	0.49	/	/	[64]
La_0.42_Y_0.28_Mg_0.3_Ni_3.5_	1.55	1.49	0.489	0.244	0.70	−32.18	31.01	
La_0.64_Sm_0.15_Mg_0.20_Ni_3.56_Al_0.15_	1.28	/	0.009	0.005		−33.9	35.3	[68]
La_0.62_Sm_0.16_Y_0.02_Mg_0.20_Ni_3.56_Al_0.15_	1.32	/	0.008	0.004	/	−32.3	34.6	
La_0.58_Sm_0.18_Y_0.01_Mg_0.20_Ni_3.62_Al_0.16_	1.39	/	0.030	0.025	/	−24.4	26.4	[98]
La_0.58_Sm_0.21_Y_0.01_Mg_0.23_Ni_3.62_Al_0.16_	1.35	/	0.036	0.030	/	−25.9	29.2	
La_0.72_Y_0.13_Mg_0.15_Ni_3.65_Al_0.15_Mn_0.05_	1.38	/	0.007	0.006	/	−29.7	25.4	[99]
La_0.72_Y_0.13_Mg_0.15_Ni_3.65_Al_0.15_Fe_0.05_	1.44	/	0.014	0.006	/	−22.9	24.8	
La_0.74_Y_0.22_Mg_0.04_Ni_3.338_Al_0.132_	1.352		0.281	0.187	0.407	−27.10	25.24	[87]
La_0.63_Y_0.33_Mg_0.04_Ni_3.722_Al_0.198_	1.381		0.226	0.155	0.377	−28.29	26.31	
La_0.52_Y_0.44_Mg_0.04_Ni_3.206_Al_0.264_	1.449	/	0.188	0.139	0.302	−29.22	27.57	
LaY_2_Ni_10.95_Mn_0.45_	1.64	0.56	0.14/0.04	0.09/0.03	0.41/0.43	/	32.66/33.87	[89]
La_0.85_Ce_0.15_Y_2_Ni_10.95_Mn_0.45_	1.65	1.06	0.18/0.07	0.15/0.05	0.22/0.44	/	32.06/31.96	
La_0.25_Ce_0.75_Y_2_Ni_10.95_Mn_0.45_	1.56	1.08	0.33	0.33	0.56	/	27.38	
La_0.3_Mg_0.7_Ni_2.8_Co_0.5_	1.64	1.46	0.027	0.014	0.65	/		[102]
La_0.75_Mg_0.25_Ni_3.3_Co_0.2_	1.14	/	0.04	0.027	0.4	/		[103]
Mm_0.83_Mg_0.17_Ni_2.89_Al_0.17_Co_0.2_Fe_0.05_	1.39	1.23	0.0146	0.0116	0.23	−33	/	[43]

## Data Availability

No new data were created or analyzed in this study. Data sharing is not applicable to this article.
